# Infection-induced lysine lactylation enables herpesvirus immune evasion

**DOI:** 10.1126/sciadv.ads6215

**Published:** 2025-01-08

**Authors:** Matthew D. Tyl, Victoria U. Merengwa, Ileana M. Cristea

**Affiliations:** Department of Molecular Biology, Princeton University, Lewis Thomas Laboratory, Washington Road, Princeton, NJ 08544, USA.

## Abstract

Aerobic glycolysis is a hallmark of many viral infections, leading to substantial accumulation of lactate. However, the regulatory roles of lactate during viral infections remain poorly understood. Here, we report that human cytomegalovirus (HCMV) infection leverages lactate to induce widespread protein lactylation and promote viral spread. We establish that lactyllysine is enriched in intrinsically disordered regions, regulating viral protein condensates and immune signaling transduction. Dynamic lactylation of immune factors suppresses immunity, a feature we show to be shared for HCMV and herpes simplex virus 1 infections, through regulation of RNA binding protein 14 and interferon-γ–inducible protein 16 (IFI16). K90 lactylation of the viral DNA sensor IFI16 inhibits recruitment of the DNA damage response kinase DNA-PK, preventing IFI16-driven virus gene repression and cytokine induction. Together, we characterize global protein lactylation dynamics during virus infection, finding that virus-induced lactate contributes to its immune evasion through direct inhibition of immune signaling pathways.

## Teaser

Virus induction of a Warburg-like effect promotes lactylation of immune response proteins to prevent initiation of host immunity.

## INTRODUCTION

Aerobic glycolysis is a hallmark of many viral infections (*1*). This metabolic alteration promotes cellular proliferation during normal physiology and across pathologies, most notably during cancer (*2*). Glycolytic up-regulation necessarily produces large amounts of lactate, which has recently become recognized as a key feature of this metabolic phenotype through suppression of local inflammatory immune cells (*3*). Viruses similarly have acquired mechanisms to evade host immunity to support their replication and spread, however, the contribution of lactate to viral replication and immune evasion remains underexplored.

Human cytomegalovirus (HCMV), a widely spread and oncomodulatory β-herpesvirus, induces marked alterations to cellular metabolism, beginning with and ultimately driven by an increase in glycolysis as part of a Warburg-like effect (*4*–*7*). As an obligate parasite, HCMV relies on the host cell to provide precursors for its proteins, genome, and lipid envelope. HCMV infection enables this through global transformation of host protein abundances (*8*), stability (*9*), spatial organization (*10*), protein-protein interactions (*11*), and posttranslational modification (PTM) state (*12*). Although energy production and anabolic pathways are known to fuel production of new virus particles, it remains unclear whether HCMV-induced metabolic dysregulation indirectly supports cell-to-cell virus spread. Beyond metabolic regulation of immune cells, Zhang *et al.* demonstrated that lactate induces the addition of a PTM on lysine residues, resulting in lactyllysine as an epigenetic histone modification (*13*). Lactyllysine additionally decorates the cellular proteome, altering metabolism (*14*–*17*), DNA repair (*18*, *19*), and immune cell proliferation and differentiation (*20*, *21*). However, lactyllysine-mediated regulation of the cellular proteome has not been explored during human virus infections. Furthermore, direct regulation of immune signaling proteins by lysine lactylation may enable immune evasion across pathologies, including virus infection, but has not yet been explored.

Herpesviruses, including HCMV, rapidly disable host immune signaling capacities to allow for virus replication and dissemination. Upon depositing the viral DNA into the nucleus to begin viral gene expression and genome replication, the viral genome is recognized by the interferon-γ–inducible protein 16 (IFI16), which stimulates cytokine expression in part via stimulator of interferon genes (STING) through the TANK-binding kinase 1 and interferon regulatory factor 3 (TBK1-IRF3) axis and directly suppresses herpesvirus transcription (*22*–*24*). IFI16 additionally exhibits cross-talk with other viral DNA sensors, such as cytosolic DNA cyclic guanosine monophosphate–adenosine monophosphate synthetase (cGAS) (*25*), while also intersecting with the DNA damage response (DDR) upon recognition of DNA damage or foreign DNA (*26*–*29*). Herpesvirus proteins disrupt host innate immunity through combinatorial inhibition across these pathways. HCMV pUL83 prevents IFI16 aggregation (*30*). ICP0 of herpes simplex virus 1 (HSV-1), a virally encoded E3 ubiquitin ligase, targets IFI16 and antiviral components of the DDR pathways, such as DNA-dependent protein kinase (DNA-PK), for degradation (*31*). HSV-1 pUL37, HCMV pUL31, and Kaposi’s sarcoma-associated herpesvirus ORF52 all directly inhibit cGAS (*32*–*34*), whereas HCMV pUL82 further inhibits STING to prevent antiviral immune signaling (*35*).

As immune signaling pathways must always be present to launch antiviral immunity, they must be kept inactive outside of infection to prevent autoimmunity. In particular, immune signaling proteins are exquisitely controlled by PTMs. cGAS is highly modified by different PTM types (*36*, *37*). IFI16 phosphorylation has been shown to promote its liquid-liquid phase separation (LLPS) (*38*), a required step for immune induction, whereas downstream interferon (IFN) production relies on a phosphorylation signaling cascade through TBK1 and IRF3 (*39*). The cell additionally encodes mechanisms to lock pathways in an inactive state during planned nucleic acid stress, such as V(D)J recombination during B cell maturation or the S phase of the cell cycle (*28*). Virus infection could leverage host-programmed inactivation mechanisms to further promote fitness.

Here, we find that infection-induced lactate promotes the spread of HCMV in human fibroblasts. HCMV-induced lactate accumulation leads to a global increase in the host cell proteome lactylation. We use lactyllysine enrichment paired with mass spectrometry (MS) to investigate pathways dynamically regulated by lactylation throughout virus replication. First, we find an enrichment of lactylation within protein intrinsically disordered regions (IDRs), a feature conserved across disease states and in *Escherichia coli*. Computational motif analysis and treatment with alanine suggest a contribution of alanyl-tRNA synthetase (AlaRS) enzymes as lactyltransferases (*17*, *40*) targeting protein IDRs and affecting virus spread. IDR lactylation on the viral protein pUL112, a structural component of the HCMV genome replication compartment through its LLPS behavior (*41*), disrupts its interaction with the DNA polymerase subunit pUL44. Further investigating the host lactylome, we find that immune response proteins are highly lactylated throughout infection. Mutagenesis and functional assays uncover a proviral function for the RNA binding protein 14 (RBM14), as well as a role for its K600 lactyl-like modification on promoting HCMV spread and inhibiting innate immunity. By similarly profiling global lactylation upon infection with HSV-1, we find that the up-regulation of lactylation on immune signaling pathways is a conserved feature for both HCMV and HSV-1 infections. Functional analyses demonstrate that IFI16 K90 lactylation, a modification conserved across both herpesvirus infections, is proviral for these viruses through inhibition of IFI16 functions in viral gene suppression and cytokine signaling. IFI16 is known to induce antiviral immunity by intersecting with the DDR pathways (*26*, *27*). We find that IFI16 K90 lactyl-mimic interferes with its ability to recruit active DNA-PK to incoming viral DNA, thus inhibiting host immune responses to virus infection. Together, this study reveals that herpesvirus-induced metabolic remodeling leverages the lactyllysine signaling axis, disrupting immune signaling transduction to promote virus spread.

## RESULTS

### HCMV infection induces protein lactylation en route to promoting viral spread

**Fig. 1.**
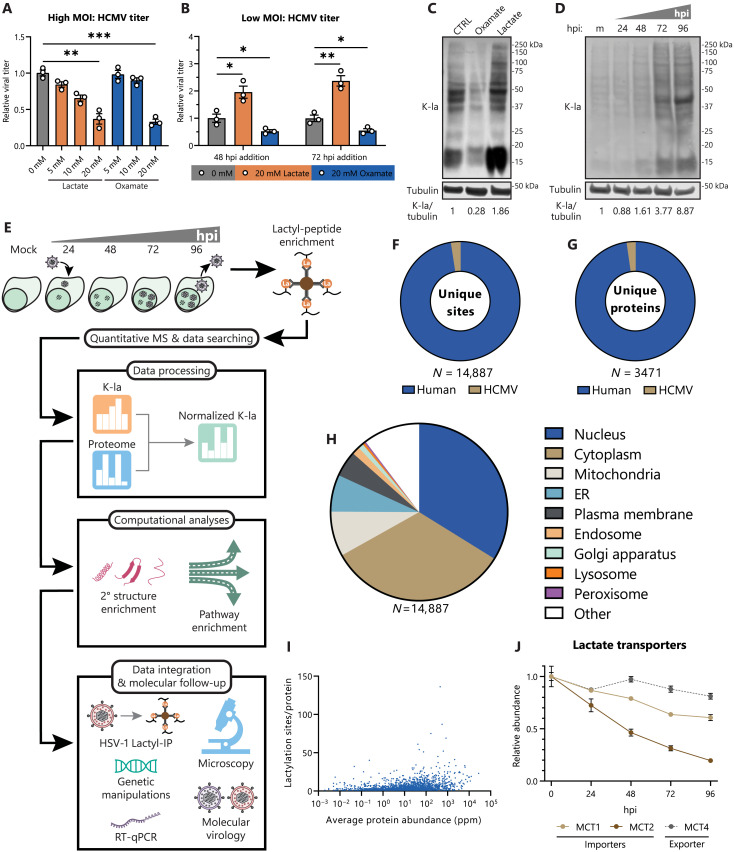
HCMV-induced lactate causes widespread proteome lactylation and promotes virus spread. (**A**) Virus titer after HCMV (strain TB40/E) infection of WT MRC-5 fibroblasts (MOI 1, 120 hpi, *n* = 3). Media were untreated (0 mM) or supplemented with 5 to 20 mM sodium lactate or sodium oxamate. Replicates were normalized by the untreated average virus titer. (**B**) Virus titer as in (A), except MOI 0.01, 12 dpi. Media were swapped from control media to untreated (0 mM), 20 mM lactate, or 20 mM oxamate media at the indicated hpi and then incubated until collection at 12 dpi. (**C**) MRC-5 cells were infected with HCMV (MOI 3) and treated with 0 mM, 20 mM lactate, or 20 mM oxamate. Cells were incubated until 96 hpi, followed by immunoblot analysis of whole-cell lysate with pan anti-lactyllysine antibody (K-la) or anti-tubulin (loading control). (**D**) MRC-5 cells were mock infected (m) or infected with HCMV (MOI 3) and collected at the indicated hpi and then processed as in (C). (**E**) Workflow for global lactylome sample preparation and subsequent data processing, computational analysis, and functional follow-up. RT-qPCR, reverse transcription qPCR. (**F**) Number of unique lactyllysine sites identified across (**G**) unique host and viral proteins in the HCMV global lactylome dataset. (**H**) Primary subcellular localization (UniProt) of lactylated protein across all lactylation sites. ER, endoplasmic reticulum. (**I**) Number of identified lactylation sites as a function of absolute protein abundance in parts per million (ppm) sourced from PaxDb, *H. sapiens*–lung (integrated). (**J**) Protein abundances of the primary lactate importers and exporters during infection with HCMV, *n* = 3. Data are representative of three independent experiments [(C) and (D)]. Bar plots are means ± SEM, with significance determined by two-tailed Student’s *t* test [(A) and (B)]. *P < 0.05, **P < 0.01, and ***P < 0.001.

HCMV is known to markedly alter the cellular metabolism, including an increase in glycolytic flux via a Warburg-like effect (*5*). Notably, as an enveloped virus, carbon flux toward lipid synthesis pathways is highly up-regulated (*42*). However, most of the pyruvate produced by glycolysis is excreted as lactate, accumulating to a greater extent than in uninfected cells (fig. S1A). To determine how lactate alters virus replication, we tested increased and decreased lactate levels by treating with lactate and the lactate dehydrogenase inhibitor, oxamate, respectively. We considered both the effect of lactate on a single virus replication cycle and on cell-to-cell virus spread. To test a single virus replication cycle, we infected human lung fibroblasts (MRC-5) at a high multiplicity of infection (MOI of 1), thereby inducing simultaneous infection of most cells. After a single virus replication cycle [120 hours after infection (hpi)], either increasing or decreasing lactate levels resulted in decreased virus titer (Fig. 1A), which is not due to cytotoxic levels of lactate or oxamate (fig. S1C). This observation suggests that dysregulating the levels or temporality of lactate production affects virus replication. To test the effect of lactate on cell-to-cell spread, we infected cells at a low MOI (0.01), allowing for multiple rounds of virus replication prior to collection at 12 days after infection (dpi). Prior reports demonstrated that increased flux through glycolysis is evident at 48 hpi (*42*), leading to lactate accumulation and secretion at 48 to 72 hpi (*43*). Taking this temporality into account, we tested the impact of lactate levels by delayed treatment of infected cells at 48 and 72 hpi. At both time points, lactate increased virus titer, whereas oxamate decreased titer (Fig. 1B). These findings point to a complex relationship between HCMV and lactate metabolism, whereby high lactate can promote virus spread if it follows a specific temporality of lactate accumulation. Furthermore, the decrease in titer during a single replication cycle, contrary to the supplementation of other carbon sources (*44*), suggests that lactate may act through a nonmetabolic mechanism to increase virus spread. Lactate was recently shown to covalently modify lysine residues, leading to lysine lactylation or lactyllysine (*13*). We find that manipulation of lactate abundance through oxamate or lactate supplementation leads to a decrease or increase in global protein lactylation during infection, respectively (Fig. 1C). In agreement with the kinetics of lactate accumulation during infection, global protein lactylation increases by 72 hpi (Fig. 1D).

To discover what proteins, host or viral, become temporally lactylated during infection, fibroblasts were collected at time points spanning early (24 hpi), delayed early (48 hpi), and late (72 and 96 hpi) stages of virus replication, in conjunction with an uninfected (mock) sample for normalization (Fig. 1E). Identification and temporal quantification of specific lactyllysine sites was accomplished by immunoaffinity purification of lactyl-peptides (lactyl-IP) using pan anti-lactyllysine antibodies, prior to analysis by MS. In parallel, an unenriched sample was used for determination of proteome dynamics throughout infection. Biological replicates displayed high reproducibility, with *R* > 0.80 for all lactyl-peptide enriched replicates and *R* > 0.95 for all proteome replicates (fig. S1, D and E). Upon optimizing the IP buffer and duration for the lactyl-IP protocol, we enhanced the enrichment efficiency, obtaining 30% of peptide-spectrum matches (PSMs) for lactyl-peptides (fig. S1F). In total, ~15,000 lactyllysine sites were identified across ~3500 host and viral proteins (Fig. 1, F and G). These lactyllysine sites modified proteins across major subcellular compartments (Fig. 1H), potentially enacting cell-wide regulatory events.

Lactyllysine identifications were not limited to highly abundant proteins. When accounting for absolute protein abundances in lung tissue (PaxDb), lactylations during infection of MRC-5 lung fibroblasts were identified on proteins ranging from 0.002 to 27,557 parts per million (ppm) (Fig. 1I). Although variation in lactyl-peptide abundance is only modestly accounted for by protein abundance variation (*R*^2^ < 0.45 in all time points) (fig. S1G), lactyl-peptide dynamics were normalized to changes in the modified protein abundance to identify lactyllysine sites with regulated stoichiometry throughout infection. Together, these metrics demonstrate that our temporal lactylation dataset provides the necessary depth and reproducibility to determine dynamically modified pathways throughout infection and discover functional protein lactylation sites that alter virus replication.

Independent analysis of the proteomic changes during infection showed a substantial decrease in major lactate importers, monocarboxylate transporters 1 and 2 (MCT1 and MCT2), with only modest changes to the lactate exporter MCT4 (Fig. 1J and table S1). This likely contributes to the observed net outward flux of lactate during HCMV infection (*6*, *42*). Notably, as HCMV has been shown to increase the susceptibility of proximal uninfected cells to subsequent infections (*45*), the regulatory roles of lactate are poised to extend beyond the infected cell to neighboring cells. The secretion of lactate into the microenvironment may also function to temper any antiviral impacts of lactate accumulation within the infected cell.

### Lactyllysine displays an evolutionarily conserved enrichment in protein IDRs

**Fig. 2.**
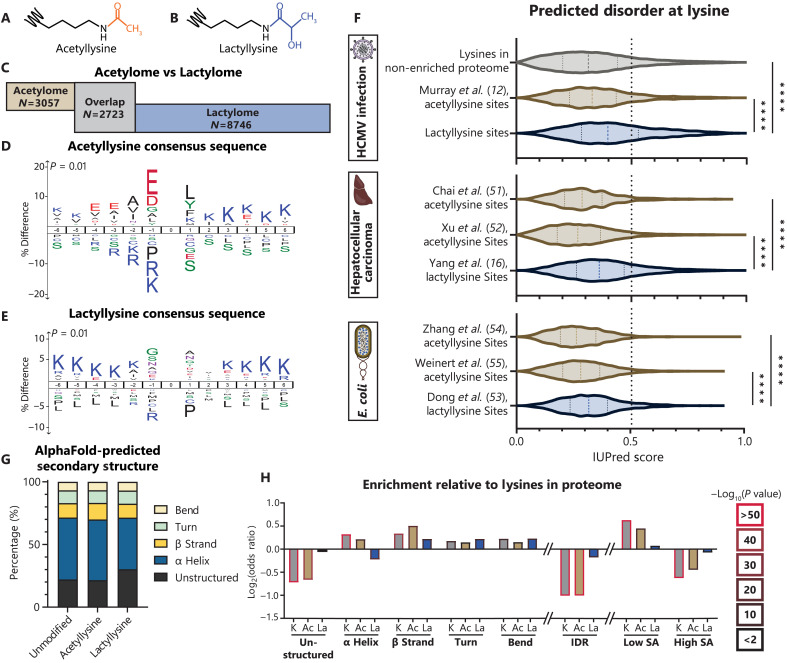
Evolutionarily conserved enrichment of lactyllysine in protein IDRs. (**A** and **B**) Schematic of acetyllysine and lactyllysine. (**C**) Unique acetyllysine, unique lactyllysine, or overlapping sites by comparison to the HCMV-infected MRC-5 acetylome (*12*). (**D**) Acetyllysine consensus sequence or (**E**) lactyllysine consensus sequence determined with iceLogo. (**F**) IUPred3-predicted intrinsic disorder score across the depicted datasets (*12*, *16*, *51*–*55*). (**G**) Proportion of each dataset with lysines in the indicated AlphaFold-predicted secondary structure group (**H**) with statistical enrichment compared to the theoretical human proteome, including predicted solvent accessibility (SA). Dotted lines on violin plots separate quartiles (F). Significance determined by two-tailed Student’s *t* test. ****P < 0.0001.

PTMs occur across protein structural motifs, toggling the local biochemistry to regulate protein function. For example, phosphorylation in short unstructured regions can open an enzyme catalytic site, called a kinase activation loop (*46*). Lysine acetylation (Fig. 2A) can occur on lysine-rich nuclear localization signals within protein IDRs or within DNA binding domains constructed from α helices (*47*, *48*). By comparison, the regulatory functions of lysine lactylation (Fig. 2B) on nonhistone proteins require further characterization. Through integration of a previously generated global acetylation dataset during HCMV infection of fibroblasts from our group (*12*), we found acetylation and lactylation to comodify 2723 lysine sites. However, 3057 acetylation sites and 8746 lactylation sites were observed on nonoverlapping residues (Fig. 2C). Hence, despite some overlap, lactylation modifies a distinct set of lysines in the proteome as compared to acetylation. This distinction is also evident when considering the motifs found to contain these modifications. Although acetyllysine shows strong consensus with negatively charged glutamate and aspartate residues at the −1, −3, and −4 positions (Fig. 2D), lactyllysine is enriched in lysine-rich primary sequences with small and polar amino acids in the −1 and +1 positions, such as glycine, alanine, serine, threonine, and asparagine (Fig. 2E). This lactyllysine consensus sequence agrees with prior global lactylation datasets (*16*, *21*, *40*). The enrichment of lysines may be contributed by the well-known bias resulting from trypsin protein digestion; however, it is noteworthy that this enrichment is maintained even when comparing to other proteome and acetylome studies that similarly used trypsin.

The unique amino acid environment for these PTMs indicates that they may preferentially modify distinct protein secondary structures. For example, the residues observed in lactyllysine motifs are frequently found in IDRs (*49*). Using IUPred3, a state-of-the-art tool for predicting protein IDRs from local amino acid sequence (*50*), we predicted the disorder score at each acetylation or lactylation site, as well as for all unmodified lysines in the unenriched proteome acquired in this study. Lactyllysine sites have a significantly higher median IUPred score (0.40) than the unmodified lysines (0.31) or acetyllysines (0.33) (Fig. 2F). By mining other datasets, we found this trend to be conserved, including in human liver tissue afflicted by hepatocellular carcinoma (*16*, *51*, *52*) and in a different kingdom of life, *E. coli* (*53*–*55*). This observation was further validated by overlaying our datasets onto the high-confidence predicted secondary structures produced by AlphaFold2 using StructureMap (*56*, *57*). An enrichment of lactyllysine in unstructured regions of proteins was found, concomitant with a decrease in representation of α helices (Fig. 2G); whereas unmodified lysines and acetyllysines are significantly less likely to be in unstructured regions or IDRs relative to all lysines in the theoretical proteome, this is not true for lactyllysine (Fig. 2H). In addition, through integration of our data with disordered regions from known protein structures annotated in InterPro (*58*), we find that lactyllysine is identified more frequently in disordered regions than lysines in the unenriched proteome or acetyllysines (fig. S2B). The lower coverage of IDRs in the unenriched proteome may partly derive from the bias of using trypsin for protein digestion, which cleaves after lysine and arginine residues that are often clustered within IDRs (*49*). Tryptic peptides resulting from IDRs may be too small or not unique, thereby impeding their identification in database searching. The underrepresentation of unstructured regions is also observed for acetyllysine, whereas lactyllysine occurs in unstructured regions or IDRs frequently enough to overcome this bias. Furthermore, whereas identified unmodified lysines and acetyllysines are less likely to occur in high solvent accessibility (SA; Fig. 2H) regions, lactyllysine matches the SA frequency of the theoretical proteome. This suggests that our global lactylation dataset is rich in regulatory PTMs as SA is frequently a predictor of functional PTM sites (*59*).

### AARS1 induces IDR lactylation to promote virus spread

Our finding that lysine lactylation shows an evolutionarily conserved enrichment in protein IDRs likely derives from the biochemical preference of enzymes that add or remove lactylation. Enzymes reported as delactylases also function as deacetylases (HDAC1-3 and SIRT1-3) (*60*), thereby not explaining the bias in targeting protein IDRs. When considering the addition of lactylation, the identified lactyltransferases include enzymes that also add acetylation (i.e., p300, CBP, TIP60, and HBO1) (*13*, *18*, *19*, *61*, *62*) and enzymes that specifically catalyze lactylation, i.e., alanyl-tRNA synthetase enzymes (AlaRS or AARS) (*17*, *40*). The cytoplasmic AARS1 was shown to mediate global lysine lactylation (*40*), whereas AARS2 was demonstrated to catalyze mitochondrial protein lactylation (*17*). Furthermore, overexpression of the *E. coli* AlaRS in human cells was sufficient to up-regulate lysine lactylation, with the modified sites displaying a strong overlap between human and bacterial homologs (*40*). This included a similar consensus sequence to that found in our study, with glycine, alanine, serine, and asparagine at the −1 position. In addition, we find that these AlaRS-targeted sites display a high median IUPred score (0.63; fig. S2C). This also supports our observation that *E. coli* lactylation sites are enriched in predicted IDRs relative to acetylation (Fig. 2F). Hence, it is possible that AlaRS proteins have an evolutionarily conserved preference for modifying protein IDRs.

**Fig. 3.**
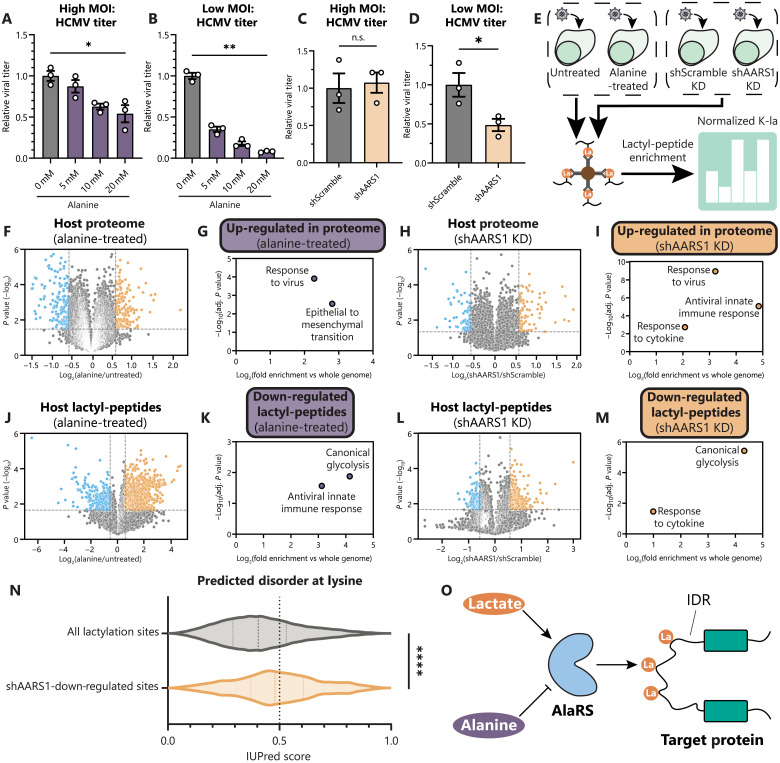
AARS1-mediated IDR lactylation regulates innate immunity. (**A**) Virus titer after HCMV infection of WT MRC-5 fibroblasts (MOI 1, 5 dpi, *n* = 3) or (**B**) (MOI 0.01, 12 dpi, *n* = 3). Media were untreated (0 mM) or treated with 5 to 20 mM alanine. Replicates were normalized by the untreated average virus titer. (**C** and **D**) Virus titer as in (A) and (B), except in MRC-5 fibroblasts with stable AARS1 KD (shAARS1) or control (shScramble). (**E**) Workflow for global lactylome sample preparation from cells infected with HCMV (96 hpi) either left untreated, treated with 100 mM alanine, expressing shScramble, or expressing shAARS1. (**F** to **I**) Volcano plots showing host protein abundances as log_2_ fold change of alanine-treated versus untreated (F) or shAARS1 versus shScramble (H) abundance during infection, with corresponding enriched GO biological processes among up-regulated proteins using g:Profiler [(G) and (I)]. (**J** to **M**) As in (F) to (I), except with host lactyl-peptide abundances, with corresponding enriched GO terms among down-regulated sites. (**N**) IUPred3-predicted intrinsic disorder scores for all lactylation sites detected compared to only down-regulated sites in shAARS1 cells. (**O**) Schematic of AlaRS-catalyzed lactylation of protein IDRs. Bar plots are means ± SEM [(A) to (D)]; dotted lines on violin plots separate quartiles (N). Significance determined by two-tailed Student’s *t* test. n.s., not significant, *P < 0.05, **P < 0.01, and ****P < 0.0001.

To assess whether these enzymes play a role in HCMV production and spread, we first treated fibroblasts with alanine, a known natural inhibitor of AARS1 (*40*). The addition of alanine causes a modest decrease in virus titer at a high MOI (Fig. 3A), similar to treatment with lactate or oxamate (Fig. 1A). However, at a low MOI, alanine treatment induces up to a 12-fold decrease in virus titer (Fig. 3B). We additionally generated cell lines with stable knockdown (KD) of AARS1 expression (shAARS1; fig. S2D). Infection of shAARS1 cells shows no difference in virus titer at a high MOI when compared to the control (Fig. 3C). However, at a low MOI, shAARS1 expression results in decreased virus titer (Fig. 3D). These findings suggest that AlaRS lactyltransferase function, particularly that of AARS1, contributes to HCMV cell-to-cell spread.

To query the impact of AlaRS functions on the proteome and lactylation state during infection with HCMV, we used two parallel approaches: (i) treatment of wild-type (WT) fibroblasts with 100 mM alanine compared to untreated cells and (ii) AARS1 KD compared to a scramble control (Fig. 3E). We found that both alanine treatment and AARS1 KD resulted in the up-regulation of proteins involved in response to a virus and the innate immune response (Fig. 3, F to I, and table S5), suggesting that the actions of AlaRS proteins normally diminish immune activation. Furthermore, we normalized lactyl-peptide abundances to changes in the proteome and then assessed which pathways showed a decrease in protein lactylation due to the inhibition of AlaRS activity or loss of AARS1. We found that alanine treatment or AARS1 KD led to down-regulated lactylation on proteins related to cytokine responses, innate immunity, and glycolysis (Fig. 3, J to M, and table S6). In addition, these shAARS1–down-regulated lactylation sites are enriched in predicted protein IDRs by IUPred3 (Fig. 3N), showing that AARS1 contributes to the enrichment of lactylation in protein IDRs. Together, these findings suggest that AARS1 suppresses immune activation, likely through lactylation of protein IDRs, establishing a critical role for AlaRS functions in the spread of HCMV infection. The prevalence of lactylation in IDRs suggests that AlaRS enzymes may specialize in different modes of protein regulation relative to other lysine PTM writers (Fig. 3O).

### Widespread HCMV protein lactylation affects processes involved in virus replication

**Fig. 4.**
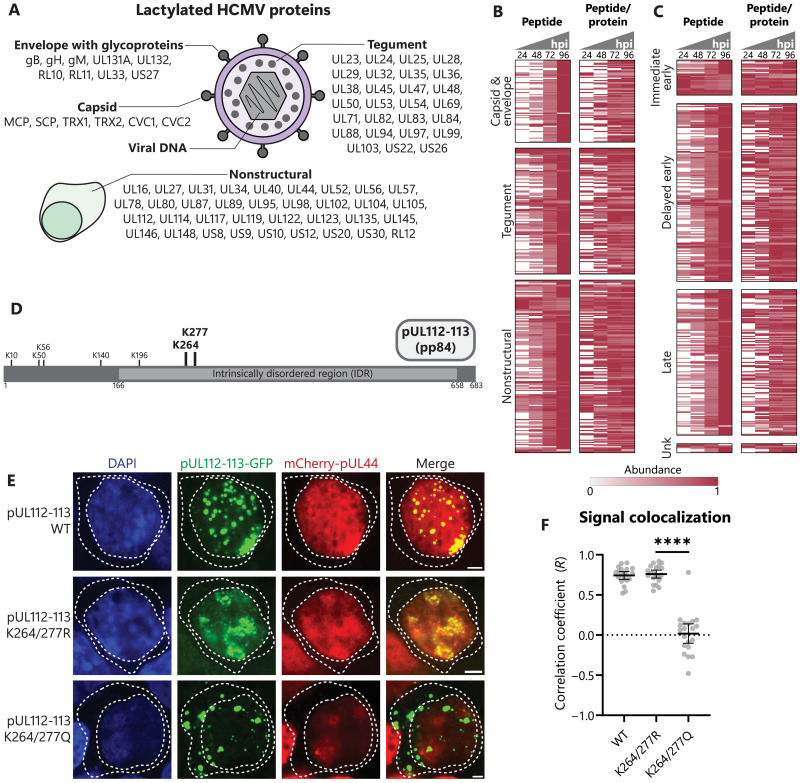
Prominent viral protein lactylation regulates processes important for replication. (**A**) Schematic of all identified lactylation sites on viral proteins separated by protein type in the virus particle or nonstructural proteins expressed during infection. (**B**) Heatmap showing temporal viral protein lactyl-peptide abundances without normalization (peptide) or normalized to abundance of that viral protein (peptide/protein) throughout infection. Proteins are grouped by virus protein type or (**C**) gene temporal class. Each peptide is normalized to maximum abundance across time points of infection. Unk, unknown. (**D**) Schematic of identified lactyllysines on the pp84 isoform of pUL112-113. (**E** and **F**) Transfection of plasmid expressing WT, K264/277R, or K264/277Q pUL112-113-GFP, along with mCherry-pUL44, in HEK293T cells. Representative images at 100× are shown (scale bars, 2.5 μm), with dotted lines bounding the nucleus and cytoplasm. Colocalization [Pearson’s correlation coefficient (PCC)] of the signal was measured within the nucleus (10 nuclei per *n*; *n* = 3). Bar plots are means ± 95% confidence interval (CI), with significance determined by two-tailed Student’s *t* test. ****P < 0.0001.

To determine the temporal regulation offered by lactylation throughout infection, we started by assessing proteins uniquely present during infection, i.e., viral proteins. Although the PTM of viral proteins still remains an understudied aspect of viral infections, accumulating evidence points to the critical contribution of host and viral enzymes to regulating virus protein function through dynamic PTMs (*63*). Our analysis revealed that an impressive number of HCMV proteins, 77 in total, become decorated with lactylation at 350 sites during the progression of infection (Fig. 4A; fig. S3, A to C; and table S2). We categorized these lactyllysine sites in two ways. First, we considered the known arrangement of viral proteins within a virus particle (capsid, envelope, and tegument) and the additional expression of nonstructural proteins during virus replication. Second, we classified the modified peptides according to the known temporal classes of viral protein expression during infection, i.e., immediate early, delayed early, and late (*4*). We find that viral protein lactylation broadly increases across all viral protein types and temporal classes throughout infection (Fig. 4, B and C). Considering that the abovementioned temporal cascade of viral gene expression also leads to a stepwise increase in protein abundance (*64*), we normalized lactylation levels to temporal viral protein abundances. Our results show that the changes in lactylation are not primarily driven by protein abundances, and site-specific viral lactylation trends are observed during infection (Fig. 4, B and C). Hence, dynamic lactylation of viral proteins occurs prominently throughout infection, being poised to regulate virus-virus and virus-host interactions.

Given the observation that lactylation occurs frequently within IDRs, our attention turned to a viral protein that is known to use its IDR for driving viral genome replication, the nonstructural protein pUL112-113 (Fig. 4D). The delayed early gene *UL112-113* encodes four phosphoproteins (pp34, pp43, pp50, and pp84) produced by alternative mRNA splicing, all of which coordinate assembly of the viral genome replication compartment (*65*). Caragliano *et al.* showed that pUL112-113 isoforms accomplish this through a LLPS mechanism dependent on their IDRs (*41*). We observed three lactylation sites within the IDR of pUL112-113, including a cluster at K264 and K277, which have not been previously found among protein acetylation sites during HCMV infection (*12*). To determine whether these lactylation sites regulate pUL112-113 functions, we generated green fluorescent protein (GFP)–tagged constructs expressing these two residues within the longest isoform (pp84) as either WT (lysine; K), charge-mimic (arginine; R), or lactyl-mimic (glutamine; Q). The pUL112-113 mutants exhibited similar expression levels (fig. S3D). As expected, microscopy analyses revealed nuclear puncta formation for the WT pUL112-113 construct (*41*). Although this localization was conserved for the K264/277R mutant, the K264/277Q pUL112-113 displayed both nuclear and cytoplasmic puncta (fig. S3, E and F). Prior studies have demonstrated that pUL112-113 uses its IDR to recruit viral DNA polymerase components through interactions with their IDRs, such as pUL44, increasing the local concentration of genome replication factors. To test whether the pUL112-113 IDR lactylation modulates such virus-virus interactions, we cotransfected our pUL112-113 constructs with mCherry-tagged pUL44. The WT pUL112-113 showed colocalization with pUL44 within nuclear puncta, which was maintained for the K264/277R mutant (Fig. 4, E and F). This colocalization is in agreement with the reported accumulation of pUL44 in pUL112 droplets. In contrast, the nuclear puncta formed by pUL112-113 K264/277Q showed poor correlation with pUL44. Hence, our results suggest that lactylation-based neutralization of the positive-charge cluster in the pUL112-113 IDR, despite not altering the size of pUL112-113 condensates (fig. S3G), obstructs the interaction with pUL44 and its inclusion into pUL112-113 condensates. This finding is also consistent with our observation that lactate decreases virus titer in a single-cycle infection (Fig. 1A). Lactylation of viral proteins by host enzymes may constitute a host-acquired strategy to dampen the fitness advantage of lactate production in promoting virus spread.

### Lactyllysine dynamically modifies metabolic and immune signaling pathways

**Fig. 5.**
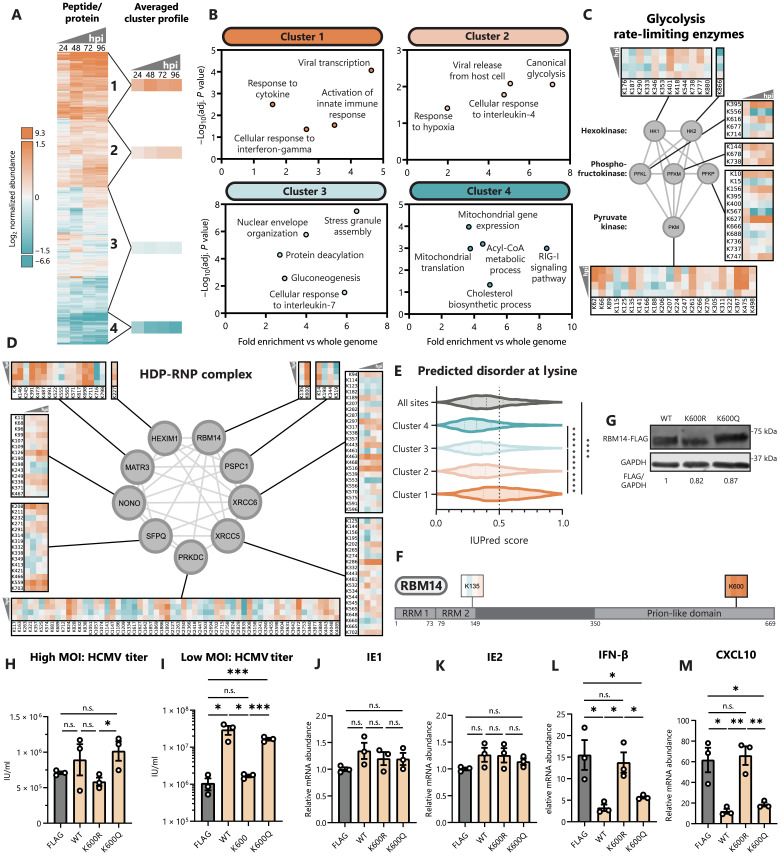
Host protein lactylation dynamics reveal RBM14 as a lactylation-regulated proviral factor. (**A**) Heatmap showing temporal host protein lactyl-peptide abundances (normalized to protein abundance) throughout infection, with hierarchical clustering into four clusters. Each peptide is shown as log_2_ fold change over mock (uninfected) abundance. hpi, hours after infection. (**B**) Scatterplots showing select enriched GO biological processes within each cluster generated using g:Profiler. (**C**) All identified lactylation sites on glycolysis rate-limiting enzymes or (**D**) on HDP-RNP complex members. Temporal abundances are normalized and scaled as in (A). (**E**) IUPred3-predicted intrinsic disorder score across the whole dataset or within individual clusters. (**F**) Schematic of identified lactylation sites on RBM14. RRM, RNA recognition motif. (**G**) MRC-5 cell lines expressing RBM14-FLAG WT, K-to-R, and K-to-Q constructs. Immunoblot analysis with anti-FLAG or anti-GAPDH (loading control). (**H**) Virus titer after HCMV infection of RBM14 or FLAG (control) cell lines at MOI 1 (5 dpi, *n* = 3) or (**I**) MOI 0.01 (12 dpi, *n* = 3). IU/ml, infectious units/ml. (**J** to **M**) *IE1*, *IE2*, *IFN-*β, and *CXCL10* mRNA levels were quantified by qPCR (ΔΔCt against GAPDH) (HCMV MOI 5, 6 hpi, *n* = 3). Replicates were normalized by the average FLAG mRNA levels [(J) and (K)] or mock mRNA levels [(L) and (M)]. Dotted lines on violin plots separate quartiles (D); bar plots are means ± SEM [(H) to (M)]. Significance determined by two-tailed Student’s *t* test. n.s., not significant, *P < 0.05, **P < 0.01, ***P < 0.001, and ****P < 0.0001.

To ascertain which host pathways are being targeted by lactylation, we performed hierarchical clustering of temporal lactyllysine levels after normalization to protein abundance (Fig. 5A and table S2). Gene ontology (GO) term enrichment of the resulting four clusters revealed specific pathways with infection-induced increases and decreases in lactylation. For example, proteins responsible for cholesterol biosynthesis processes display markedly decreased lactylation levels during infection (Fig. 5B, cluster 4). Cholesterol synthesis is known to be up-regulated during HCMV infection as cholesterol is an integral component of the viral envelope (*66*). This raises the possibility that lactylation may be removed from these proteins to maintain high pathway flux. Also down-regulated during HCMV infection is lactylation of gluconeogenesis proteins (Fig. 5B, cluster 3), whereas canonical glycolysis proteins show up-regulated lactylation sites (Fig. 5B, cluster 2), including PKM lactylation at K62 (Fig. 5C). Glycolysis has previously been shown to autoinhibit its activity by lactylation of pyruvate kinase M (PKM) at K62 (*14*). As HCMV induces a pronounced increase in glycolysis, it is possible that PKM lactylation is an activated host feedback loop to temper glycolytic flux.

Among the highly up-regulated lactylation sites during infection, proteins involved in immune signaling pathways are prominently represented (Fig. 5B, cluster 1). These proteins include those functioning in activating innate immunity, as well as the response to IFNs and other cytokines. Lactate is known to dampen immune cell effector functions (*3*), but comparatively less is known about its impact on intrinsic immunity and initiation of the innate immune response. Among the enriched CORUM complexes in cluster 1 was the HEXIM1-DNA-PK-paraspeckle components-ribonucleoprotein complex (HDP-RNP), a protein complex found to regulate immune signaling downstream of DNA sensing (*67*). We find the HDP-RNP complex to contain a multitude of highly up-regulated lactylation sites throughout infection (Fig. 5D). By analyzing the predicted secondary structures across these clusters, we find that cluster 1 lactylation sites are also most enriched in predicted protein IDRs relative to the rest of the lactylome (Fig. 5E). Cluster 1 sites display a strong enrichment of lactylation sites proximal to small, polar, and positively charged amino acids (fig. S4A), whereas cluster 4 sites show a weaker consensus sequence more closely resembling acetyllysine (Fig. 2D and fig. S4B). Our results show that lactylation is poised to modulate immune signaling pathways through modifying protein IDRs, perhaps underlying the lactate-induced support of HCMV spread.

### RBM14 K600 lactyl-mimic inhibits innate immunity

Given that our results showed that lactate promotes HCMV spread and that proteins involved in host immune responses become lactylated, we sought to understand whether these two findings are linked and, hence, to determine the effect of protein lactylation on virus spread and immune signaling. As mentioned above, numerous components of the HDP-RNP complex were lactylated during HCMV infection. A complex subunit displaying some of the highest increases in lactylation throughout infection was RBM14. An identified lactylated site on RBM14, K600, localizes within its prion-like domain, which is an IDR (Fig. 5F). When considering the previous acetylome study during HCMV infection (*12*), this K600 was not a site found to be acetylated. RBM14 is a known component of paraspeckles involved in RNA processing and DNA repair (*68*, *69*), whose function as a component of HDP-RNP remains unclear. In the context of viral infection, RBM14 RNA processing functions are important for latent viral gene expression for both HIV and Epstein-Barr virus (*70*, *71*), and its function was also shown to support influenza replication (*72*). However, RBM14 has not been characterized during a lytic DNA virus infection.

To characterize the effect of RBM14 K600 lactylation, we constructed fibroblasts stably expressing WT, charge-mimic (K-to-R), and lactyl-mimic (K-to-Q) versions of RBM14 (Fig. 5G). We confirmed equivalent expression levels of these constructs, as well as their expected nuclear localization (fig. S5A). To assess the impact on virus production and spread, we tested infections with a high MOI, as well as a low MOI that allows for a multicycle infection. Although the effect on the high MOI infection was minimal for all tested constructs (Fig. 5H), a clear proviral role was seen upon expression of WT RBM14 for the low MOI experiment (Fig. 5I). This proviral effect at a low MOI was retained for the lactyl-mimic mutant (>15-fold increase in HCMV titer for K600Q) but lost for the charge-mimic mutant. Given that this effect was specifically evident for the low MOI infection, this result suggests that RBM14 lactyl-like modification at K600 supports virus spread rather than virus replication in the infected cell. To further test this possibility, we monitored both virus gene expression and immune induction. Expression of WT, K600R, and K600Q RBM14 did not alter expression of the immediately early viral genes *IE1* and *IE2* (Fig. 5, J and K). However, expression of WT and K600Q RBM14 decreased the induction of the cytokines *IFN-*β and *CXCL10* (Fig. 5, L and M), as well as the IFN-stimulated genes *ISG54* and *ISG56* (fig. S5, B and C). This suppression of cytokine levels was lost for the K600R mutant. Consistent with a role in regulating immune signaling, RBM14 was observed to interact with the viral DNA sensor IFI16 during HSV-1 infection (*73*). To test whether this is conserved during HCMV infection, we stained for RBM14 and IFI16 at 6 hpi, finding colocalization of RBM14 with IFI16 puncta (fig. S5D). Together, our results demonstrate that RBM14 inhibits immune signaling to promote viral spread, a function that is enabled by a lactyl-like modification within its IDR at K600.

### Conservation of immune signaling pathway lactylation during HSV-1 infection

**Fig. 6.**
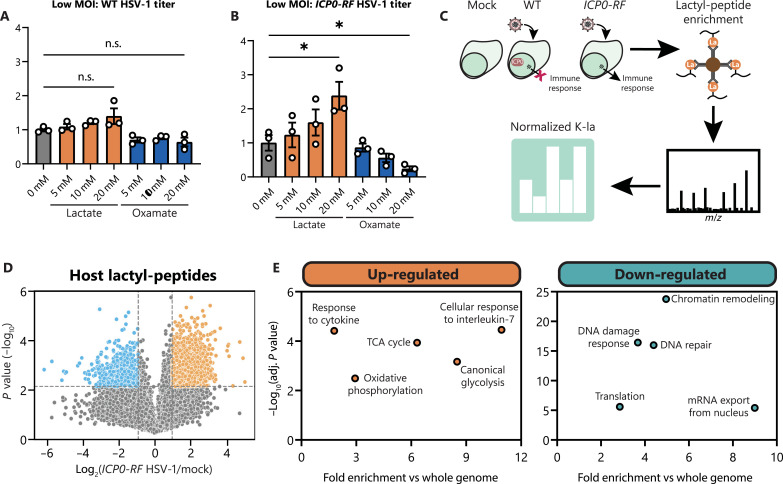
Lactate supports HSV-1 spread while lactylating immune signaling pathways. (**A**) Virus titer after WT (2 dpi, *n* = 3) or (**B**) *ICP0-RF* HSV-1 infection (3 dpi, *n* = 3) of WT MRC-5 fibroblasts at MOI 0.01. Media were untreated (0 mM) or supplemented with 5 to 20 mM sodium lactate or sodium oxamate. Replicates were normalized by the untreated average virus titer. (**C**) Workflow for global lactylome sample preparation from mock or WT/*ICP0-RF* HSV-1–infected samples (6 hpi) with subsequent data processing. (**D**) Volcano plot showing host protein lactyl-peptide abundances (normalized to protein abundance) as log_2_ fold change over mock abundance during *ICP0-RF* HSV-1 infection. Dotted lines show the threshold for differentially regulated sites: log_2_ fold change = 1 and *P* < 0.01. (**E**) Scatterplots showing select enriched GO biological processes among up-regulated or down-regulated lactyl-peptides using g:Profiler. Bar plots are means ± SEM, with significance determined by two-tailed Student’s *t* test [(A) and (B)]. n.s., not significant, *P < 0.05.

As our results indicate that lactate promotes HCMV spread by regulating immune signaling pathways, we asked whether the regulation of immune factors by lactylation is broadly relevant to virus infections. We investigated the α-herpesvirus HSV-1, a virus known to have effective host immune evasion strategies. During a low MOI infection in fibroblasts with WT HSV-1, addition of lactate or oxamate did not significantly affect virus titer (Fig. 6A), differing from WT HCMV (Fig. 1B). These viruses diverge in their metabolic programs during infection, with HSV-1 infection slightly decreasing glycolysis and lactate production during its shorter replication cycle (*74*) while also using distinct immune evasion mechanisms. HSV-1 encodes an E3 ubiquitin ligase, ICP0, which targets various host restriction factors for proteasome-dependent degradation (*31*). A mutant virus deficient in target degradation, *ICP0-RF* HSV-1, is frequently used to interrogate the DNA sensing and immune signaling pathways that are otherwise disabled by ICP0 (*26*, *75*). Performing the same low MOI infection with *ICP0-RF* HSV-1 revealed that lactate promotes virus spread in a dose-dependent manner, whereas oxamate decreases titer (Fig. 6B).

We proceeded to determine lactyl-proteome dynamics upon WT and *ICP0-RF* HSV-1 infection, focusing on a time point that we used for prior immune signaling and cytokine assays, 6 hpi (Fig. 6C). A mock (uninfected) sample was used for normalization, as well as quantification of the proteomes to allow for normalization of lactyl-peptide abundance to protein abundance. As expected, WT but not *ICP0-RF* HSV-1 infection leads to down-regulation of ICP0-targets IFI16, PML, and PRKDC in the proteome (fig. S6, C to E, and table S3). Thousands of lactylation sites were found to be either up-regulated or down-regulated upon infection with both strains of HSV-1 (Fig. 6D, fig. S6F, and table S4). Of note, similar to HCMV infection, immune signaling and metabolic pathways are increasingly lactylated during *ICP0-RF* HSV-1 infection (Fig. 6E). Furthermore, *ICP0-RF* and WT HSV-1 infections induce a reduction in lactylation on proteins involved in chromatin remodeling, DNA repair, and the DDR (Fig. 6E and fig. S6G). Despite differences in cellular metabolism during HCMV and HSV-1 infections, host factors involved in regulating immune signaling become prominently modified by lactylation during both infections. In addition, lactate appears to retain a similar effect on supporting virus spread, however only when HSV-1 is unable to suppress host innate immune factors through targeted protein degradation.

### IFI16 lactyl-mimic inhibits its antiviral functions partly via loss of active DNA-PK recruitment

**Fig. 7.**
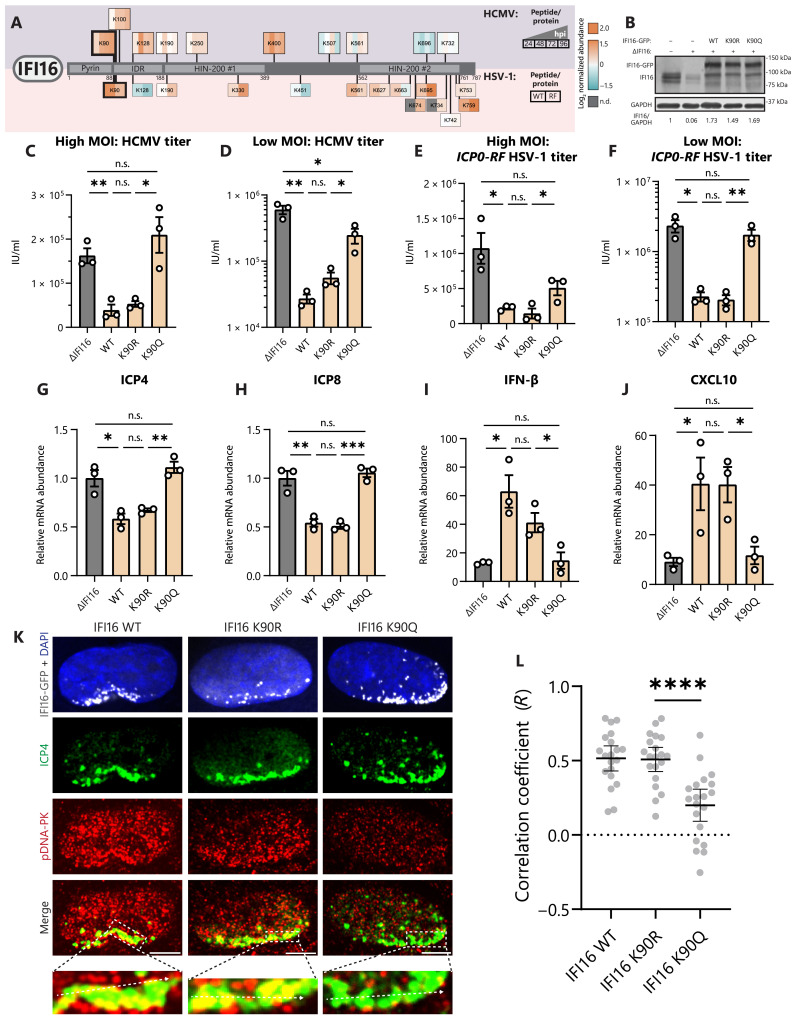
IFI16 lactylation inhibits innate immunity through loss of active DNA-PK recruitment. (**A**) Schematic of identified lactylation sites on IFI16 during infection with HCMV (top) or HSV-1 (bottom). Lactyl-peptide abundances (normalized to protein abundance) are shown as log_2_ fold change over mock (uninfected) abundance. hpi, hours after infection; WT, WT HSV-1; RF, *ICP0-RF* HSV-1; n.d., not determined. (**B**) Δ*Scramble*, Δ*IFI16*, or Δ*IFI16* HFF cell lines expressing indicated IFI16-GFP constructs. Immunoblot analysis with anti-IFI16 or anti-GAPDH (loading control) antibodies. (**C**) Virus titer after HCMV infection of IFI16 stable cell lines or Δ*IFI16* (control) (MOI 1, 5 dpi, *n* = 3) or (**D**) (MOI 0.01, 12 dpi, *n* = 3). IU/ml, infectious units/ml. (**E** and **F**) Virus titer as in (C) and (D), except with *ICP0-RF* HSV-1 infection and collection at (E) 1 dpi or (F) 3 dpi. (**G** to **J**) *ICP4*, *ICP8*, *IFN-*β, and *CXCL10* mRNA levels were quantified by qPCR (ΔΔCt against GAPDH) (*ICP0-*RF HSV-1 MOI 5, 6 hpi, *n* = 3). Replicates were normalized by the average Δ*IFI16* mRNA levels [(G) and (H)] or mock mRNA levels [(I) and (J)]. (**K** and **L**) *ICP0-RF* HSV-1–infected cell lines (MOI 5, 3 hpi) were stained for DNA-PK activation (pDNA-PK) and ICP4 expression. Representative images at 100× are shown (scale bars, 2.5 μm). Colocalization (PCC) between pDNA-PK and ICP4 was measured at the line (10 nuclei per *n*; *n* = 3). Bar plots are means ± SEM [(C) to (J)] or means ± 95% CI (L), with significance determined by two-tailed Student’s *t* test. n.s., not significant, *P < 0.05, **P < 0.01, ***P < 0.001, and ****P < 0.0001.

Despite differences in viral replication strategies, both HCMV and HSV-1 must disable many of the same host restriction factors. One of these host factors is IFI16, a viral DNA sensor that recognizes viral genomes upon their deposition into the nucleus. Upon binding to viral DNA, IFI16 both directly represses viral gene expression and activates the STING-TBK1-IRF3 axis to promote pro-inflammatory cytokine expression (*22*–*24*). Both HCMV and HSV-1 have acquired immune evasion strategies that target IFI16, with pUL83 of HCMV preventing IFI16 oligomerization and ICP0 of HSV-1 completely abolishing IFI16 functions by targeting it for degradation (*30*, *31*). We find that IFI16 is decorated by lactylation during both HCMV and HSV-1 infections (Fig. 7A). We confirm by immunoblotting that IFI16 lactylation increases upon infection with HCMV (fig. S7A). During HCMV infection, IFI16 is increasingly lactylated at multiple sites in its IDR region. Notably, it is lactylated at K128 in its nuclear localization signal, a site previously shown to be acetylated by p300 (*47*). This may contribute to the observed mislocalization of IFI16 to the cytoplasm late during HCMV infection (*76*). By contrast, during infection with either WT or *ICP0-RF* HSV-1, there are several up-regulated lactylation sites in the HIN-200 #2 domain of IFI16, perhaps interfering with its DNA binding ability. Our ability to still detect some levels of IFI16 upon WT HSV-1 infection is due to our focus on an early time point of infection.

Conserved for both HCMV and HSV-1 infections, we found an up-regulation of K90 lactylation in the protein IDR, a site that has not previously been shown to be acetylated during infection (*12*). This finding prompted us to investigate the function of this lactylation by mutagenizing this residue and generating stably expressing cell lines in a Δ*IFI16* background (Fig. 7B). IFI16 WT, K90R, and K90Q constructs showed similar expression levels and retained the expected nuclear and nucleolar localization (fig. S7B). At both high and low MOI HCMV infection, WT and K90R IFI16 restricted virus replication, whereas K90Q caused a nearly complete loss of function (Fig. 7, C and D). These trends were similar to those observed for the infection with either the Δ*UL83* HCMV strain (fig. S7, C and D) or the *ICP0-RF* HSV-1 (Fig. 7, E and F). To determine which aspects of IFI16 functions are affected by the K90 lactyl-mimic, we tested both viral gene expression and cytokine induction upon *ICP0-RF* HSV-1 infection (6 hpi). Whereas WT and K90R IFI16 restricted the viral genes *ICP4* and *ICP8* and induced robust expression of the cytokines *IFN-*β and *CXCL10*, K90Q IFI16 phenocopied ΔIFI16 (Fig. 7, G to J).

Cytokine signaling and DDR pathways are highly interlinked (*28*). For example, IFI16 recruits active DNA-PK—a master regulator of nonhomologous end joining (NHEJ) after DNA double-strand breaks (DSBs)—onto incoming viral DNA during HSV-1 infection to enable its antiviral functions (*26*, *27*). To test whether K90 lactylation affects the interaction between IFI16 and DNA-PK, we infected our IFI16 cell lines (WT, K90R, and K90Q) with *ICP0-RF* HSV-1 and visualized the active DNA-PK kinase by staining for its autophosphorylation at S2056 (pDNA-PK) (Fig. 7K). At 3 hpi, we saw clustering of IFI16 at the nuclear periphery in all cell lines. Upon staining for ICP4, a viral transcription factor that marks viral DNA, we confirmed that IFI16 localized near viral DNA in all cell lines, in agreement with its recruitment to incoming viral DNA. Hence, IFI16 relocalization and viral DNA recognition appear unperturbed by the K90 lactyl-mimic. However, whereas WT and K90R IFI16 enabled enrichment of pDNA-PK on early ICP4-labeled transcription compartments, K90Q cells showed impaired recruitment of pDNA-PK (Fig. 7L). Together, our results demonstrate that lactyl-mimic IFI16 is unable to transduce immune signaling after DNA sensing or directly restrict viral replication, in part through failure to interface with DNA-PK, an antiviral component of the DDR.

## DISCUSSION

**Fig. 8.**
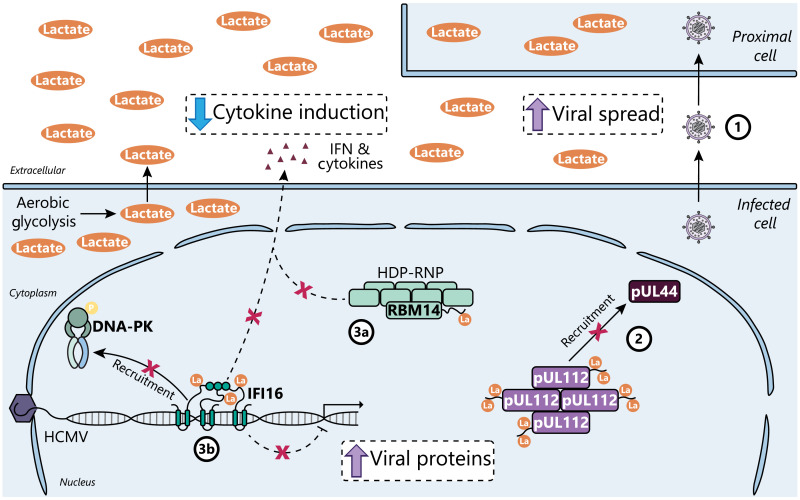
Virus-induced lactate enables immune evasion through immune signaling protein lactylation. (1) HCMV infection causes aerobic glycolysis as part of its metabolic program, which we find to promote cell-to-cell spread by lactate-induced lysine lactylation. (2) pUL112 IDR lactylation interferes with the recruitment of pUL44 into pUL112 condensates. (3a) RBM14 IDR lactylation suppresses the innate immune functions of the HDP-RNP complex, whereas (3b) IFI16 IDR lactylation inhibits coordination with DNA-PK to enact antiviral immunity, resulting in both decreased cytokine induction and increased viral protein production.

Viruses frequently induce aerobic glycolysis during infection (*1*). Although modulation of cellular metabolism is known to fuel production of virus particles, our study shows how increased lactate production and secretion indirectly increases virus fitness by promoting viral spread. Here, we find that HCMV infection up-regulates lactate production to induce widespread lysine lactylation of protein IDRs. We show evidence for functional protein lactylation both in virus replication within an infected cell and in virus propagation between cells. Lactylation of viral protein IDRs regulates viral protein-protein interactions important for formation of nuclear LLPS compartments. Although host metabolic pathways display complex site-specific regulation, immune signaling proteins are covered by highly up-regulated lactylation sites, including RBM14 and IFI16. RBM14 K600 and IFI16 K90 lactylation both promote virus spread by subverting host immunity (Fig. 8), a feature that is shared during infection with HSV-1. In addition to these functional characterizations of specific lactylation events, our study provides a rich resource of lactylated sites on both virus and host proteins during infection with two pervasive human pathogens, HCMV and HSV-1.

Among the acetyltransferase enzymes that have been characterized to catalyze lysine lactylation (*13*, *18*, *19*, *61*, *62*) are alanyl-tRNA synthetase enzymes (AlaRS/AARS) (*17*, *40*). Although the AlaRS enzymatic preference for protein sequences or structures remains mostly unexplored, our analysis suggests that AlaRS proteins target predicted protein IDRs (fig. S2C). We demonstrate that AARS1, one of two AlaRS proteins in eukaryotic cells, promotes HCMV cell-to-cell spread while also up-regulating lactylation on several innate immune proteins, including nucleolin and the IFN-induced guanosine triphosphate–binding protein Mx1 (table S6). The enzymes regulating IFI16 and RBM14 lactylation remain to be uncovered. As prior reports have demonstrated that p300 can interact with and promote the acetylation of the IFI16 IDR (*47*), it remains to be determined whether p300 can use its lactyltransferase activity to modify IFI16 during infection (*13*). However, our lactylome datasets demonstrate that AARS1 contributes to the widespread IDR lactylation that we uncover during HCMV infection, a feature that may be conserved more broadly across kingdoms of life, yet it remains unclear why this preference exists. One possibility is that the protein more efficiently recognizes lysines in regions rich with positively charged and polar amino acids, which are themselves biased toward protein IDRs (*49*). An alternative is that the enzyme requires a flexible peptide chain for docking into the active site. By analogy to its better studied function in catalyzing the addition of alanine to tRNA, although tRNA is highly ordered, the 3′ end of tRNA that accepts the alanine is not as rigid or sterically hindered (*77*), thus making IDRs more like its primary substrate. In addition, alanine can competitively inhibit protein lactylation. We find that alanine treatment inhibits HCMV cell-to-cell spread (Fig. 3B). Along with the copious secretion of lactate during infection, alanine intracellular concentration and secretion both increase during HCMV infection (*78*). Our results suggest that alanine accumulation may contribute to host defense, elevating alanine in line with lactate to temper proviral proteome lactylation by AARS1.

Innate immune signaling upon pathogen recognition and DDR pathways coordinate to induce inflammatory cytokine expression (*28*). Beyond cytosolic sensing of severe DNA damage by cGAS, DDR kinases ataxia-telangiectasia mutated (ATM) and DNA-PK can each promote inflammation after DNA DSBs (*26*, *79*). However, the homology-directed repair (HDR) kinase ATM is proviral during many DNA virus infections, aiding in viral DNA amplification and repair (*28*). Opposing its functions upon DSBs, ATM unexpectedly suppresses inflammation during HSV-1 infection by interfering with the DNA-PK–dependent DDR (*27*). Although DDR and inflammatory pathways are often linked, cellular mechanisms exist to break this link.

During the S phase of the cell cycle, cells are less sensitive to DNA damage and other inflammatory stimuli (*80*, *81*), likely serving as a tolerance mechanism for the high frequency of DNA damage during genome replication without triggering inflammation-induced hypermutation (*82*). DSB repair is biased toward HDR during S phase, using the newly synthesized homologous DNA template for “error-free” repair by the ATM pathway, and away from NHEJ repair by the DNA-PK pathway (*83*). Lactate accumulates throughout the cell cycle (*84*), promoting ATM binding to DNA by MRN complex lactylation on subunits MRE11 and NBS1 (*18*, *19*). NBS1 K388 lactylation is additionally up-regulated during HCMV infection (table S2). Notably, DNA viruses manipulate the cell cycle to gain access to the S-phase proteome, providing molecular machinery and nucleotide resources to replicate viral genomes (*4*). Stalling at the G_1_-S boundary may additionally enable virus immune evasion through lactate production.

As lactylation is a toggle for DNA repair modality, RBM14 and IFI16 lactylation may break the connection between DDR kinases and immune signaling pathways. RBM14 promotes NHEJ upon DSBs by recruiting DNA-PK (*69*). Although NHEJ is decreased during the S phase, it is still an active pathway (*83*). Lactylation of RBM14 may serve as a cell cycle–dependent signal to prevent DDR activities of DNA-PK while still promoting resolution of the DSB. Furthermore, IFI16 enables cytokine expression upon DSB in both ATM and DNA-PK pathways (*26*, *29*). However, in a context such as HSV-1 infection where ATM is not linked to cytokine expression, IFI16 is rapidly degraded by the viral protein ICP0 (*31*), allowing pATM to supplant pDNA-PK on viral DNA (*27*). We find that IFI16 lactyl-mimic is additionally sufficient to inhibit pDNA-PK binding to viral DNA (Fig. 7, K and L), allowing the cell to break the link between the DDR and cytokine expression by posttranslational inactivation of the DNA sensor IFI16. Lactylation of IFI16 K90 is conserved during HSV-1, despite HSV-1 not causing substantial changes to lactate production. Perhaps HCMV and HSV-1 infection each activate a host-programmed toggle of IFI16 by replicating at the G_1_-S phase boundary.

Although IFI16 is commonly studied during nuclear DNA virus infection, IFI16 additionally functions as a tumor suppressor through interaction with p53 (*85*). Following genotoxic chemotherapy, IFI16 can inhibit DNA repair by suppressing activation of ATM (*86*). Cancer cells have an elevated reliance on DNA repair pathways due to the tremendous replication stress present in highly proliferative cells (*87*). Lactylation of MRE11 and NBS1 promotes ATM activity and resistance to genotoxic chemotherapies (*18*, *19*), adding another layer of protumorigenic lactate function. IFI16 interferes with ATM activity, which is required for cancer cell fitness; thus, it stands to reason that IFI16 may additionally be lactylated during cancers. The K90Q lactyl-mimic mutation used in this study has been previously identified in lung squamous cell carcinoma from the COSMIC database (cancer.sanger.ac.uk) (*88*). Breaking the link between the DDR and immune signaling may be a host-programmed, cell cycle–dependent mechanism that is overactivated to allow tumorigenesis and continued immune evasion.

HCMV itself is an oncomodulatory virus, being connected to increased malignancy of tumors, with its Warburg-like metabolic reprogramming expected to underlie this phenomenon (*7*). HCMV infection not only reprograms the infected cell but also primes nearby cells for infection (*45*). Neighboring cells show high levels of nonstructural and tegument viral proteins, correlating with an accumulation of cells in mitosis and early S phase, as well as a diminished immune response. Because of the pronounced lactate secretion during infection, cells proximal to the primary infection center likely exist in a lactate-rich environment, similar to the tumor microenvironment (*3*). The combination of viral proteins and elevated lactate in nearby cells could break the link between the DDR and immune signaling to promote virus spread, but overactivation of ATM may also increase the risk of oncomodulation. Persistent lactate accumulation alone is sufficient to cause mitotic slippage, promoting cancer cell survival (*84*). This suggests that virus-induced lactate production may contribute to HCMV-induced oncomodulation, a possibility that should be explored in future studies.

## MATERIALS AND METHODS

### Experimental model and subject details

#### Cell culture conditions

WT MRC-5 cells [American Type Culture Collection (ATCC) CCL-171, passage numbers 16 to 26], Δ*IFI16* human foreskin fibroblast (HFF) cells (a gift from J. Justice, passage numbers 12 to 18) (*26*), human embryonic kidney (HEK) 293T cells (ATCC CRL3216), and Phoenix-Ampho cells (ATCC CRL-3213) were cultured in high-glucose Dulbecco’s modified Eagle’s medium (DMEM) (Sigma-Aldrich) supplemented with 10% fetal bovine serum (FBS) (Gemini Bio-Products, 100-106) and 1% (v/v) penicillin-streptomycin solution (Gibco) at 37°C in 5% CO_2_ (standard growth media). Cells were tested for mycoplasma using the MycoStrip (InvivoGen) Mycoplasma Detection Kit and were authenticated by cell morphology and growth curve analyses.

#### Virus strains and infections

WT HCMV TB40/E, Δ*UL83* HCMV AD169 mutant (*30*), WT HSV-1 17^+^ (a gift from B. Sodeik, Hannover Medical School, Hannover, Germany), and *ICP0-RF* HSV-1 mutant (a gift from B. Roizman, University of Chicago, Chicago, IL, USA and S. Silverstein, Columbia University, New York, NY, USA) were propagated as previously described (*75*), aliquoted, snap frozen, and stored at −80°C. Briefly, working stocks were generated from P_0_ stocks by infecting U-2 OS cells (HSV-1 strains), MRC-5 cells (WT HCMV TB40/E), or Δ*IFI16* HFF cells (Δ*UL83* HCMV AD169) at a low MOI (~0.01 to 0.001 plaque-forming units per cell). Infections were allowed to propagate for ~2 to 4 days (HSV-1 stains) or ~12 to 14 days (HCMV strains), before supernatants and infected cells were collected. Cell-associated virus was released from cells by sonication, pooled with the supernatant, and then subjected to ultracentrifugation [20,000 rpm, 2 hours, 4°C with a SW28 swinging bucket rotor (Beckman Coulter)] over a 20% sorbitol cushion to concentrate virus. Virus stock titers were determined by plaque assay on U-2 OS (HSV-1 strains) or TCID_50_ (median tissue culture infectious dose) on MRC-5 (HCMV strains) monolayers.

Infections were conducted at the indicated MOI in DMEM supplemented with 2% FBS (HSV-1 strains) or 10% FBS (HCMV strains) with intermittent rocking at 37°C and 5% CO_2_. The inoculum was removed after 1 hour, and media were replaced with standard growth media. Unless otherwise indicated, cells remained in this media for the duration of the experiment. For the purposes of our study, 0 hpi is considered to be 60 min after addition of the inoculum to account for the adsorption of the virus particles.

#### Media treatments

WT MRC-5 cells were infected with the indicated virus strain and MOI, and then after inoculation, the media were replaced with growth media supplemented with sodium l-lactate (Sigma-Aldrich), sodium oxamate (Thermo Fisher Scientific), or l-alanine (Thermo Fisher Scientific). During immunoblotting and proteomic experiments, media were swapped every 24 hours. During virus titering experiments, media were not replaced unless indicated.

#### Lactate quantification

Cell culture media were collected from mock-infected or HCMV-infected cells after 96 hours of incubation. Media were centrifuged through a 3-kDa molecular weight cutoff column to remove protein, and then lactate was quantified using a Lactate Assay Kit (Sigma-Aldrich; MAK064) according to the manufacturer’s instructions.

#### TUNEL assay

Cells were plated at 50% confluency in 96-well plates. WT MRC-5 cells were treated with the indicated small molecule and concentration, except for all stable cell lines, which were left untreated. Cells were incubated for 96 hours, and then cell death was detected using the In Situ Cell Death Detection Kit, TMR Red (Sigma-Aldrich) according to the manufacturer’s instructions.

#### Lactyl-peptide enrichment and sample preparation

For analysis using a Q Exactive HF mass spectrometer, six to eight 15-cm dishes per sample of MRC-5 cells were washed twice with ice-cold phosphate-buffered saline (PBS), scraped into PBS, and combined into a microcentrifuge tube. Cells were spun down at 250*g*, washed with PBS, and flash frozen in liquid nitrogen until ready to be processed. Cell pellets were resuspended in a preheated lysis buffer [50 mM tris-HCl (pH 8), 100 mM NaCl, 0.5 mM EDTA, and 5% SDS] and heated at 95°C for 5 min at a time. The cell suspension was cup horn sonicated for 30 pulses and heated until all clumps were dispersed. Cell lysates were reduced and alkylated in 25 mM TCEP [tris(2-carboxyethyl)phosphine] and 50 mM chloroacetamide at 95°C for 5 min, and then the protein was isolated with methanol/chloroform precipitation. Protein disks were resuspended in 25 mM Hepes (pH 8.2) by sonication, the protein concentration was determined by BCA assay (Pierce), and then solutions were diluted to 0.5 mg/ml. Proteins were digested with MS Grade Trypsin Protease (Pierce) by two additions at 1:200 trypsin:protein and incubated at 37°C with gentle rocking for 4 and 12 hours, respectively. The digested samples were acidified to 1% trifluoroacetic acid (TFA), incubated on ice for 15 min, and then spun down at 4000*g* for 10 min at 4°C to remove the undigested protein. The supernatants were subjected to Oasis Column cleanup (Waters) per the manufacturer’s instructions. The samples were dried down by SpeedVac, and then the ~15-mg peptide was resuspended in a peptide IP buffer [50 mM Mops/NaOH (pH 7.2), 10 mM Na_2_HPO_4_, and 50 mM NaCl) at ~10 mg/ml and adjusted to pH 7 to 8. After setting aside 50 μg of peptides for whole proteome analysis, the remainder of the sample was subjected to anti-lactyllysine IP using 30-μl conjugated agarose beads per sample (PTM BIO). IP was performed for 2 hours at 4°C, and beads were washed three times each with a buffer and high-performance liquid chromatography–grade water and then eluted by three rounds of 50-μl 0.1% TFA at room temperature (RT) for 5 min. Lactyl-peptide IP and whole-cell proteome samples were adjusted to 1% TFA and subjected to C18 StageTip Desalting (Empore) cleanup and then dried down by SpeedVac and resuspended in 1% formic acid (FA) and 1% acetonitrile (ACN). 2 μg of the whole-cell proteome sample was analyzed, whereas ~1/2 of the total lactyl-peptide IP sample was analyzed. Liquid chromatography (LC)–MS-grade solvents are used beginning at trypsin digestion and maintained throughout sample injection and LC.

For analysis using a timsTOF Ultra, all conditions were kept consistent, except that one 15-cm dish of MRC-5 cells was used; ~2 mg of the peptide was resuspended in an IP buffer; 15-μl anti-lactyllysine conjugated agarose beads were used per sample; samples were resuspended in 0.1% FA and 4% ACN; and 150 ng of the peptide was analyzed for whole-cell proteome analysis.

#### Peptide LC-MS/MS analysis

*Q Exactive HF MS analysis.* Samples were analyzed on a Q Exactive HF mass spectrometer (Thermo Fisher Scientific) equipped with an EASY-Spray ion source (Thermo Fisher Scientific). Peptides were resolved for nano-LC (nLC)–tandem MS (MS/MS) using an UltiMate 3000 nRSLC system (Dionex) equipped with an EASYSpray C18 column (2 μm by 75 μm by 50 cm; Thermo Fisher Scientific). Peptides were separated with a 150-min gradient using a mobile phase composed of solvents A (0.1% FA in water) and B (0.1% FA and 2.9% water in 97% ACN) at a flow rate of 250 nl/min with a continuous gradient from 3 to 35% B over 150 min. For HCMV-infected lactyllysine immunoaffinity purification samples, an MS1 survey scan was performed from 350 to 1800 mass/charge ratio (*m*/*z*) at 120,000 resolution with an automatic gain control (AGC) setting of 3 × 10^6^ and a maximum inject time (MIT) of 30 ms. Data-dependent acquisition (DDA) MS2 scans of the top 10 ions followed each MS1 scan at 30,000 resolution with an AGC setting of 1 × 10^5^, an MIT of 150 ms, an isolation window of 1.6 *m*/*z*, a fixed first mass of 100 *m*/*z*, a minimum intensity threshold of 1 × 10^5^, peptide matching set to preferred, a loop count of 10, dynamic exclusion of 45.0 s, and acquired in centroid. The MS2 acquisition settings for whole-cell proteome samples differed in the following ways: Data-independent acquisition (DIA) scans were performed with a resolution of 30,000, an MIT of 50 ms, an AGC setting of 3 × 10^6^, and an isolation window of 24 *m*/*z*.

*timsTOF Ultra MS analysis.* Samples were analyzed on a timsTOF Ultra mass spectrometer (Bruker) equipped with a Captive Spray 2 ion source (Bruker) containing a 10 μm emitter (Bruker). Peptides were resolved for nLC-MS/MS using a nanoElute 2 nLC system (Bruker) equipped with a PepSep C18 column (1.5 μm by 75 μm by 25 cm; Bruker). Peptides were separated with a 40-min gradient using a mobile phase composed of 0.1% FA as solvent A and 0.1% FA/99.9% ACN as solvent B. A linear gradient was run consisting of 3 to 34% buffer B at a flow rate of 200 nl/min.

The HSV-1–infected lactyl-peptide enriched samples were used to generate spectral libraries in DDA-parallel accumulation serial fragmentation (PASEF) mode with five PASEF ramps. Trapped ion mobility spectrometry (TIMS) settings were at a 100-ms ramp and accumulation time (100% duty cycle) and a ramp rate of 9.42 Hz. Singly charged precursors were filtered out, and only precursor signals over an intensity threshold of 500 arbitrary units were picked for fragmentation. Precursors over the target value of 20,000 arbitrary units were dynamically excluded for 0.4 min. Precursors below 700 *m*/*z* were isolated with a 2 *m*/*z* window, whereas precursors above 800 *m*/*z* were isolated with a 3 *m*/*z* window. All spectra were acquired within an *m*/*z* range of 100 to 1700 and an ion mobility (IM) range from 1.45 to 0.60 V cm^−2^. Collision energy was decreased from 59 eV at 1/*K*_0_ = 1.6 V cm^−2^ to 20 eV at 1/*K*_0_ = 0.6 V cm^−2^.

Analysis of HSV-1–infected whole-cell proteome and lactyl-peptide enriched samples were conducted using DIA-PASEF (*89*). For lactyl-peptide enriched samples, TIMS settings were at a 100-ms ramp time and a 9.42-Hz ramp rate; for whole-cell proteome samples, this was adjusted to a 50-ms ramp time and a 17.80-Hz ramp rate. Each method includes three IM windows per DIA-PASEF scan with variable isolation widths adjusted to the precursor densities using py_diAID, with a total of 16 DIA-PASEF scans per cycle. All spectra were acquired within an *m*/*z* range of 300 to 1300, with the same IM range and collision energy parameters as the DDA-PASEF scans.

#### Peptide identification and quantification

*HCMV-infected lactyl-peptide enrichment data.* For processing of raw instrument files, MS/MS spectra were analyzed by Proteome Discoverer 2.4 (PD; Thermo Fisher Scientific) using a FASTA file containing human and herpesvirus protein sequences (UniProt-SwissProt, downloaded 2022-10) and common contaminants. Mass accuracy was recalibrated offline using the spectrum files RC node, and the Minora feature detection node was used for label-free MS1 quantitation. Sequest was run with a full tryptic search and maximum two missed cleavages, a precursor mass tolerance of 4 ppm, and a fragment mass tolerance of 0.02 Da. Included modifications were static cysteine carbamidomethylation, dynamic lysine lactylation, dynamic asparagine deamidation, dynamic methionine oxidation, dynamic N-terminal acetylation, and dynamic methionine excision at the N terminus. Percolator filtered PSMs to 1% false discovery rate. ptmRS was used to calculate localization probability for lactyl modifications, requiring a minimum score threshold of 75% confidence. Raw abundances values were exported from PD.

Lactyl-peptide raw abundances were required to be detected in two of three replicates and a coefficient of variation (CV) < 100% in at least one time point. Peptides passing these criteria were then divided by the median for variation in loading and then by the mean across all samples to center at a value of 1. Missing values for host peptides were then imputed using missForest (*90*), whereas viral peptide abundance values were not imputed. Before any molecular follow-up experiments on specific lactyllysine sites, the presence of a diagnostic cyclic immonium ion of lactyllysine at 156.103 *m*/*z* was verified in spectra of interest, indicating high-confidence lactyllysine identification (*15*).

*Whole-cell proteome data.* MS/MS spectra from HCMV-infected samples were analyzed using DIA-NN 1.8 (*91*), whereas spectra from HSV-1–infected samples were analyzed using DIA-NN 1.82 beta 27. MS/MS spectra were searched using a FASTA containing human and herpesvirus protein sequences (UniProt-SwissProt, downloaded 2022-10 for Q Exactive HF data or 2024-03 for timsTOF Ultra data) and common contaminants using trypsin as the digestion enzyme. Included modifications were static cysteine carbamidomethylation, dynamic methionine oxidation, dynamic N-terminal acetylation, and dynamic methionine excision at the N terminus. A maximum of one missed cleavage and one variable modification were allowed. Default settings were used for both searches. The mass accuracy was set at 10 ppm, the MS1 accuracy at 12 ppm, and a scan window of 8. “Match between runs” and “Heuristic protein inference” were enabled. The proteomics output tables were filtered for a maximum of 1% *q* value at both precursor and global protein levels. Two peptides per protein were required, with only unique or razor peptides used for quantification. Processing of raw abundance values was performed similarly as for the lactyl-peptide enriched data, except that viral protein abundances were also imputed using missForest.

*HSV-1–infected lactyl-peptide enrichment data.* An experimental lactyl-peptide spectral library was generated from the DDA runs on the HSV-1–infected lactyl-peptide enriched samples using FragPipe 21.1. Extracted lactyl-peptide spectra from the experimental spectral library and a FASTA containing human and herpesvirus protein sequences (UniProt-SwissProt, downloaded 2024-03) were used to generate an in silico lactyl-peptide spectral library with two missed cleavages and two variable modifications (lactyllysine variable modification was added: “UniMod:378, 72.011 Da, K”) in DIAN-NN 1.82 beta 27. Using this spectral library, we analyzed the DIA runs using DIA-NN 1.82 beta 27 with the same settings as the whole-cell proteome searches, with the following exceptions: Two missed cleavages were allowed, two dynamic modifications were allowed, dynamic lysine lactylation was added, and dynamic methionine oxidation was removed (to limit search space). We then required a “PTM.Site.Confidence” of at least 0.95 for any identified lactyl-peptides. We filtered separately for the WT/mock and *ICP0-RF*/mock comparisons, requiring peptide detection in at least one of three replicates for both infected and mock conditions, prior to data normalization and imputation using missForest.

*HCMV-infected alanine-treated or shAARS1 data.* Whole proteome and lactyl-peptide enriched data were acquired on a timsTOF Ultra and searched using DIA-NN 1.82 beta 27 as with the HSV-1–infected samples. We filtered separately for the alanine-treated/untreated and shAARS1/shScramble comparisons, requiring peptide detection in at least two of three replicates, prior to imputation using missForest. Data were not normalized according to median value to preserve differences in global lactylation abundance between the samples.

#### Motif analysis

Peptide sequences were extended using PEPTIDEXTENDER (https://schwartzlab.uconn.edu/pepextend) with the *Homo sapiens* proteome to obtain peptide sequences with six amino acids flanking either site of the lactylated, acetylated, or unmodified lysine (position 0). The extended sequences were analyzed with iceLogo (https://iomics.ugent.be/icelogoserver/) using the *H. sapiens* proteome reference set, the percentage difference scoring method, and a *P* value cutoff of 0.01 (*92*).

#### Predicted secondary structure analyses

For IUPred analyses, peptide sequences are extended to 33 amino acids using PEPTIDEXTENDER, with 16 amino acids flanking either side of the modified or unmodified lysine. IUPred long disorder score is calculated using IUPred3 (https://iupred3.elte.hu/). The window for IUPred predictions is 21 amino acids (amino acid ± 10 amino acids), so the amino acid IUPred scores for the interior 13 amino acids are only influenced by the true primary sequence. Scores for the interior 13 amino acids are averaged to give a cumulative IUPred score for that modified or unmodified lysine region, which is repeated across all relevant lysines in the datasets. For predictions based on AlphaFold structures, we used the StructureMap package in Python (*57*). For integration of disordered regions from known protein structures, we integrated annotations from InterPro (*58*).

#### Hierarchical clustering and overrepresentation analyses

Lactylated peptides during HCMV infection were clustered by log_2_ abundance normalized to protein abundance and mock infection abundance. Hierarchical clustering was conducted using the SciPy package in Python with the dendrogram cut at four clusters. For determination of enriched GO terms, pathways, and protein complexes, we used g:Profiler (https://biit.cs.ut.ee/gprofiler/gost) (*93*).

For lactylated peptides during HSV-1 infection, we thresholded up-regulated or down-regulated sites after normalization to protein abundance and mock infection abundance. Lactyl-peptides with a fold change of ±1.5 compared to mock and *P* < 0.05 across biological replicates were considered up-regulated or down-regulated during WT HSV-1 infection, whereas a fold change of ±2 and *P* < 0.01 were considered up-regulated or down-regulated during *ICP0-RF* HSV-1 infection. g:Profiler was similarly used for enrichment analysis on the differentially regulated lactyl-peptides.

#### Construct and lentivirus generation

WT, K-to-R (charge-mimic), and K-to-Q (lactyl-mimic) sequences and corresponding protein tags were cloned into the indicated vectors: *UL112-113-mEGFP*, *RBM14-3xFLAG*, and *IFI16-mEGFP* were cloned into the pLXSN retroviral vector; *UL44* was cloned into the pmCherry-C1 transfection vector. To produce lentivirus from pLXSN vectors, Phoenix-Ampho cells were plated in 6-cm dishes and transfected at ~60% confluency with 10 μg of the plasmid and 30 μl of X-tremeGENE (Sigma-Aldrich) in Opti-MEM (Gibco). Media were replaced at 6 hours after transfection (hpt) with DMEM supplemented with 20% FBS. The cell culture supernatant was collected at 48 and 72 hpt, pooled, and passed through a 0.45-μm filter.

For stable KDs, 4 μg of a pLKO.1 vector expressing a scramble target sequence (shScramble) or *AARS1* targeting sequence (shAARS1 #1: GCAGAATAAGATGTCCAACTA, #2: CGATGTCCAGAAACGAGTGTT, and #3: CCCAGGCAACATGAAGGATAA) were transfected along with 3 μg of psPAX2 and 2 μg of pMD2.G as above into HEK293T cells at ~80% confluency and then harvested as above. After verification of shAARS1 #2 as the optimal target sequence, it was used for all downstream experiments.

#### Lentivirus transductions and plasmid transfections

For lentivirus transductions to generate stably expressing transgenic cell lines, lentivirus-containing media were supplemented with polybrene (8 μg/ml; EMD Millipore) and used to transduce MRC-5 or HFF cells at 30% confluency. Cells were allowed to recover for 2 days before selection with G418 (400 μg/ml; Thermo Fisher Scientific) for pLXSN vectors until ~100% of cells contained the selectable marker.

For transient plasmid transfections for immunofluorescence experiments, HEK293T cells were seeded onto coverslips in a 12-well plate. At ~80% confluency, 3 μg of pLXSN-*UL112-113-GFP* vectors with or without 100 ng of pmCherry-C1-*UL44* were mixed with 30 μl of X-tremeGENE in Opti-MEM and added to cells for 6 hours, and then media were replaced with normal growth media. At 24 hpt, cells were fixed in 4% paraformaldehyde for 15 min at RT and washed with PBS.

#### Virus titering

Cell culture media from HCMV infections were collected at the indicated time point, the cellular debris was pelleted by centrifugation at 9000*g*, and then supernatants were used for titering; cell culture media and infected cells from HSV-1 infections were collected at the indicated time point and freeze thawed three times to extract cell-associated virus, the cellular debris was pelleted by centrifugation at 9000*g*, and then supernatants were used for titering. Virus was diluted appropriately (1:2 to 1:1000 depending on the assay) and used to infect a reporter plate of confluent MRC-5 fibroblasts. The infection was allowed to proceed for 4 hours for HSV-1 samples or 24 hours for HCMV samples, and then plates were washed with cold PBS and fixed with prechilled methanol at −20°C for 15 min. After washing out methanol with PBS, plates were blocked with 3% bovine serum albumin (BSA) in PBS with 0.2% Tween 20 (PBST) for 30 min, HCMV sample reporter plates were incubated with anti-IE1 [mouse, 1:100; clone 1B12, gift from T. Shenk (*94*)] in block, whereas HSV-1 sample reporter plates were incubated with anti-ICP4 (mouse, 1:1000; Santa Cruz Biotechnology, sc-69809) in block overnight at 4°C. Plates were washed 3× with PBST, incubated with goat anti-mouse immunoglobulin G (IgG) (H+L) highly cross-adsorbed Alexa Fluor 488 (1:1000; Thermo Fisher Scientific, A28175) and Hoechst 33342 [1:500 of a stock (1 mg/ml); Thermo Fisher Scientific] in block for 1 hour at RT, and washed 3× with PBST. An Operetta imaging system (PerkinElmer) was used to visualize the reporter plate and count the IE1-positive or ICP4-positive cells across each well, which was used to calculate infectious units/ml (IU/ml). Each sample was analyzed in technical duplicate.

#### SDS-PAGE and immunoblotting

MRC-5 or HFF cells (4 × 10^5^) were lysed in a lysis buffer (as previously) and protein quantified by BCA assay (Pierce), and then samples were prepared in a Protein Sample Loading Buffer (LICORbio) with 50 mM dithiothreitol and boiled at 95°C for 5 min. For all polyacrylamide gel electrophoresis (PAGE), samples were electrophoresed on freshly prepared 10% acrylamide Tris/glycine SDS-PAGE gels using a NuPage MES SDS Running Buffer (Thermo Fisher Scientific). Proteins were then electroblotted onto a 0.45-μm polyvinylidene difluoride membrane (EMD Millipore Immobilon), reactivated with methanol, and blocked with 5% BSA in TBS with 0.1% Tween 20 (TBST) for 30 min. Membranes were incubated overnight at 4°C with the primary antibodies diluted into block: anti–l-lactyllysine (rabbit, 1:1000; PTM BIO, PTM-1401), anti-AARS1 (rabbit, 1:1000; ProteinTech, 17394-1-AP), anti-IFI16 (mouse, 1:1000; Santa Cruz Biotechnology, SC-8023), anti-FLAG (mouse, 1:1000; Sigma-Aldrich, F1804), anti–α-tubulin (mouse, 1:5000; Sigma-Aldrich, T6199), or anti–glyceraldehyde-3-phosphate dehydrogenase (GAPDH) (rabbit, 1:5000; Cell Signaling Technology, 5174S). After washing 3× with TBST, membranes were incubated for 1 hour at RT with the secondary antibodies diluted into block: goat anti-rabbit IgG (H+L) Superclonal recombinant Alexa Fluor 680 (1:10,000; Thermo Fisher Scientific, A27042), goat anti-mouse IgG (H+L) highly cross-adsorbed Alexa Fluor Plus 800 (1:10,000; Thermo Fisher Scientific, A32730), or goat anti-rabbit IgG (H+L) Peroxidase AffiniPure horseradish peroxidase (HRP) (1:10,000; Jackson ImmunoResearch Laboratories, 111-035-003). Blots were washed 3× with TBST and imaged. Anti–l-lactyllysine blots were imaged using HRP secondary antibodies with ECL Prime reagents (Cytiva) with x-ray film, whereas all other blots were imaged using fluorescent secondary antibodies on the Odyssey DLx Imaging System (LICORbio). The signal was quantified and normalized to loading control [α-tubulin or GAPDH) in Image Studio 6.0 (LICORbio).

#### IFI16-GFP IP

For analysis of IFI16 lactylation abundance by immunoblotting, IFI16-GFP cells were left uninfected or infected with HCMV at MOI 3 and then collected by scraping into cold PBS. Cell pellets were lysed in a buffer consisting of 20 mM Hepes-KOH (pH 7.4), 110 mM potassium acetate, 2 mM MgCl_2_, 0.1% Tween 20, 1 μM ZnCl_2_, 1 μM CaCl_2_, 1% Triton X-100, 200 mM NaCl, 1:1000 universal nuclease, and 1:100 Halt protease and phosphatase inhibitor cocktail. Samples were lysed for 30 min on ice with regular vortexing, the insoluble debris was pelleted by centrifuging at 10,000*g* for 10 min at 4°C, and the protein concentration of the soluble fraction was determined by BCA. IP samples were generated by aliquoting 500 μg of each sample and bringing to a total volume of 500 μl with a lysis buffer (total protein concentration of 1 mg/ml).

Before use in the IP, 20 μl of GFP-Trap magnetic beads (ProteinTech) were washed 3× with a lysis buffer. IP was performed for 1 hour at 4°C with rotation, beads were collected using a magnetic strip, and then beads were washed 3× with a lysis buffer and 2× with water. Proteins were eluted from the beads by heating in 100 μl of a TEL buffer [106 mM tris-HCl, 141 mM Tris base, 2% lithium dodecyl sulfate (LDS), 0.5 mM EDTA] for 10 min at 70°C with shaking and then processed for immunoblotting as above.

#### RNA isolation and quantitative reverse transcription PCR

RNA extraction was performed from 2 × 10^5^ MRC-5 or HFF cells using the RNeasy Mini Kit (Qiagen) as per the manufacturer’s instructions. Then, cDNA was generated from 1 μg of RNA using the High-Capacity cDNA Reverse Transcription Kit (Thermo Fisher Scientific) as per the manufacturer’s instructions. Gene-specific primers (below) and the SYBR green PCR master mix (Life Technologies) were used to quantify cDNA by quantitative polymerase chain reaction (qPCR) on the QuantStudio 7 Flex Real-Time PCR System (Applied Biosystems). Relative mRNA quantities were determined using the ΔΔCt method with GAPDH as an internal control. Each sample was analyzed in technical triplicate. Primers for qPCR were as follows: IFN-β, 5′-GCATTACCTGAAGGCCAAGG-3′ (forward) and 5′-AAGCAATTGTCCAGTCCCAGA-3′ (reverse); CXCL10, 5′-ACGCTGTACCTGCATCAGCA-3′ (forward) and 5′-TTGATGGCCTTCGATTCTGG-3′ (reverse); IE1, 5′-AGCGCC-GCATTGAGGA-3′ (forward) and 5′-CAGACTCTCAGAGGATC-GGCC-3′ (reverse); IE2, 5′-GAGCCCGACTTTACCATCCA-3′ (forward) and 5′-CAGCCGGCGGTATCGA-3′ (reverse); ICP4, 5′-GCGTCGTCGAGGTCGT-3′ (forward) and 5′-CGCGGAGA-CGGAGGAG-3′ (reverse); ICP8, 5′-GTGGTTACCGAGGGCTTC-AA-3′ (forward) and 5′-GTTACCTTGTCCGAGCCTCC-3′ (reverse); GAPDH, 5′-TTCGACAGTCAGCCGCATCTTCTT-3′ (forward) and 5′-CAGGCGCCCAATACGACCAAATC-3′ (reverse); ISG54, 5′-ACGGTATGCTTGGAACGATTG-3′ (forward) and 5′-AACCCAGAGTGTGGCTGATG-3′ (reverse); and ISG56, 5′-AAGGCAGGCTGTCCGCTTA-3′ (forward) and 5′-TCCTGTCCT-TCATCCTGAAGCT-3′ (reverse).

#### Immunofluorescence microscopy and analysis

MRC-5 or HFF cells were seeded onto coverslips at 2 × 10^5^ cells in a 12-well plate and infected or treated as indicated. Cells were fixed in 4% paraformaldehyde for 15 min at RT and washed with PBS. MRC-5, HFF, or transfected HEK293T cells were permeabilized in 1 × PBS with 0.1% Triton X-100 (PBT) for 15 min at RT and then blocked in 5% human serum and 5% goat serum in PBT for 30 min. Samples were incubated overnight at 4°C with the primary antibody diluted into block: anti-ICP4 (mouse, 1:500; Santa Cruz Biotechnology, sc-69809) to mark viral transcription compartments, anti-DNA-PK pS2056 (rabbit, 1:500; Abcam, Ab18192) to measure DNA-PK kinase activity, anti-FLAG (mouse, 1:500; Sigma-Aldrich, F1804), or anti-RBM14 (rabbit, 1:500; Novus Biologicals, NBP1-84416). After primary staining, cells were washed 3× with PBT and then incubated for 1 hour at RT with Hoechst 33342 [1:500 of a stock (1 mg/ml); Thermo Fisher Scientific] and the appropriate secondary antibody in block: goat anti-mouse IgG (H+L) highly cross-adsorbed Alexa Fluor 488 (1:2000; Thermo Fisher Scientific, A28175), goat anti-rabbit IgG (H+L) cross-adsorbed Alexa Fluor 568 (1:2000; Thermo Fisher Scientific, A-11011), or goat anti-mouse IgG (H+L) highly cross-adsorbed Alexa Fluor 633 (1:2000; Thermo Fisher Scientific, A-21052). Cells were washed 3× with PBT and mounted using a ProLong Diamond Antifade (Thermo Fisher Scientific). Coverslips were imaged at the Princeton Confocal Imaging Core using an inverted fluorescence confocal microscope (Nikon Ti-E) equipped with a Yokogawa spinning disc (CSU-21) and digital CMOS (complementary metal-oxide semiconductor) camera (Hamamatsu ORCA-Flash TuCam) using a Nikon 100× Plan Apo objective with a ×100 magnification (single slices centered around the nuclear center) or a Nikon 60× Plan Apo objective with a ×60 magnification (maximum intensity projections) as indicated.

Image analysis was performed using ImageJ. For determination of the nuclear localization percentage of pUL112-113-GFP, integrated pixel density within a nucleus [region of interest (ROI) defined by 4′,6-diamidino-2-phenylindole (DAPI) staining] was divided by the integrated pixel density of the whole cell (ROI defined by the GFP signal) from a maximum intensity projection. For determination of the nuclear colocalization of pUL112-113-GFP and mCherry-pUL44, the nucleus ROI was defined by DAPI staining, and then Coloc 2 performed signal correlation across the nucleus ROI. For determination of the pUL112-113 particle size, the GFP signal was thresholded and particle sizes were quantified using Analyze Particles. For determination of pDNA-PK colocalization with ICP4, pDNA-PK and ICP4 fluorescence intensity profiles across a line covering ICP4 puncta in the nuclear periphery were background subtracted and compared with Pearson’s correlation.

#### Quantification and statistical analysis

Data processing and large-scale analyses were performed using Python 3.8 in conjunction with Pandas, NumPy, SciPy, Scikit-learn, Seaborn, and StructureMap libraries. Cytoscape was used to generate interaction networks. Microscopy images were analyzed in ImageJ 2.9. Statistical analysis was performed using GraphPad Prism 10. Significance was determined by two-tailed Student’s *t* test (*n* = 3 biological replicates) unless otherwise stated. Where applicable, *P < 0.05, **P < 0.01, ***P < 0.001, and ****P < 0.0001. Figures were constructed in Adobe Illustrator.
