# Supplementary material for: Infection-induced lysine lactylation enables herpesvirus immune evasion
Source: Sci Adv. 2025 Jan 8;11(2):eads6215. doi: 10.1126/sciadv.ads6215 (PMC11708889; doi:10.1126/sciadv.ads6215)
Supplement: Supplementary file 1 — Figs. S1 to S7 Legends for tables S1 to S7 [file sciadv.ads6215_sm.pdf]

Science Advances
AAAS

Supplementary Materials for

Infection-induced lysine lactylation enables herpesvirus immune evasion

Matthew D. Tyl *et al.*

Corresponding author: Ileana M. Cristea, icristea@princeton.edu

Sci. Adv. **11**, eads6215 (2025)
DOI: 10.1126/sciadv.ads6215

The PDF file includes:

Figs. S1 to S7
Legends for tables S1 to S7

Other Supplementary Material for this manuscript includes the following:

Tables S1 to S7

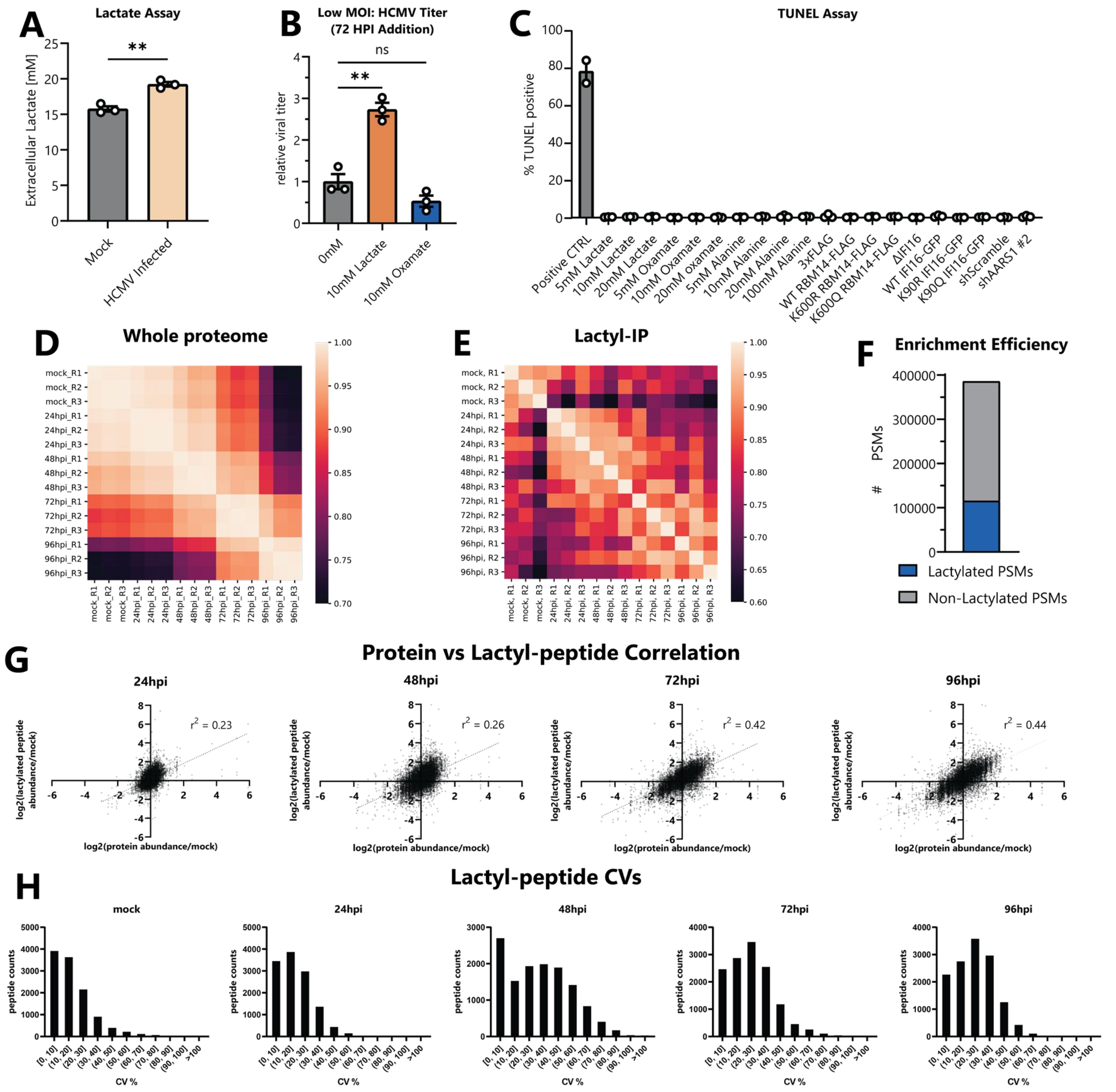

Fig. S1. HCMV-induced lactate causes widespread proteome lactylation and promotes virus spread. (**A**) Quantification of lactate in cell culture media from uninfected WT MRC-5 fibroblasts (Mock) or HCMV Infected fibroblasts at 96 hours post-infection. (**B**) Virus titer after HCMV infection of fibroblasts [multiplicity of infection (MOI) 0.01, 12 days post-infection (DPI), $n = 3$]. Media was swapped from control media to untreated (0 mM), 10 mM lactate or 10 mM oxamate media at 72 hours post-infection (HPI), then incubated until collection at 12 DPI. Replicates were normalized by the untreated average virus titer. (**C**) Cell death assay by TUNEL staining for all small-molecule treatments and stable cell lines in the manuscript [$n = 3$]. (**D**) Correlation matrix (Pearson's R) of whole cell proteome samples. (**E**) Correlation matrix (Pearson's R) of lactyl-IP samples. (**F**) Bar graph of number of peptide-spectrum matches (PSMs) across lactyl-IP samples containing an identified lactyllysine site vs unmodified peptides (~30% of spectra had an identified lactyllysine site). (**G**) Scatter plots of R^2 of lactyl-peptide abundance vs abundance of the modified protein (normalized to mock abundances) in each timepoint of infection. (**H**) Bar charts showing

lactyl-peptide abundance CVs across three biological replicates in each timepoint of infection. Bar plots are mean ± SEM (A and B). Significance determined by two-tailed Student's *t* test. ns = not significant, $^{**}P < 0.01$.

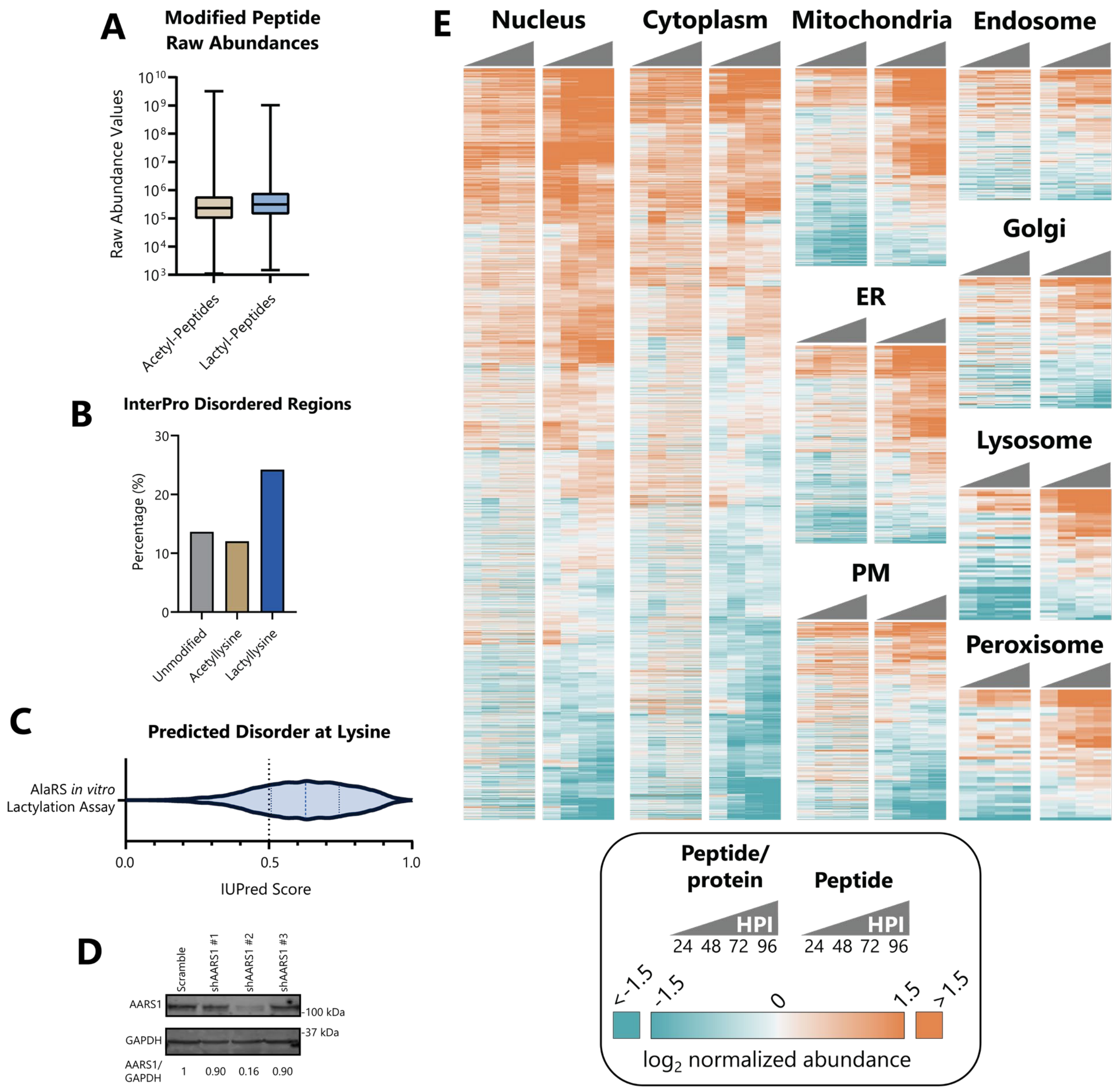

Fig. S2. Proteome lactylation dynamics and their regulation across subcellular compartments during HCMV infection. (**A**) Raw abundance values of acetylated peptides (*12*) vs lactylated peptides from the HCMV temporal dataset. (**B**) Percent of unmodified lysines, acetyllysines (*12*), or lactyllysines from this study found within annotated disordered regions of known protein structures from InterPro. (**C**) IUPred3-predicted disorder score of identified AlaRS target sites from an *in vitro* lactyltransferase assay (*40*). (**D**) MRC-5 cells expressing a Scramble shRNA or shRNA's targeting AARS1. Immunoblot with anti-AARS1 or anti-GAPDH (loading control). shAARS1 #2 was used for all subsequent assays. (**E**) Heatmap showing temporal host protein lactyl-peptide abundances with normalization to protein abundance (Peptide/protein) or without normalization (Peptide) throughout infection. Each peptide is shown as $\log_2$ fold-change

over mock (uninfected) abundance; HPI = hours post-infection; ER = endoplasmic reticulum; PM = plasma membrane.

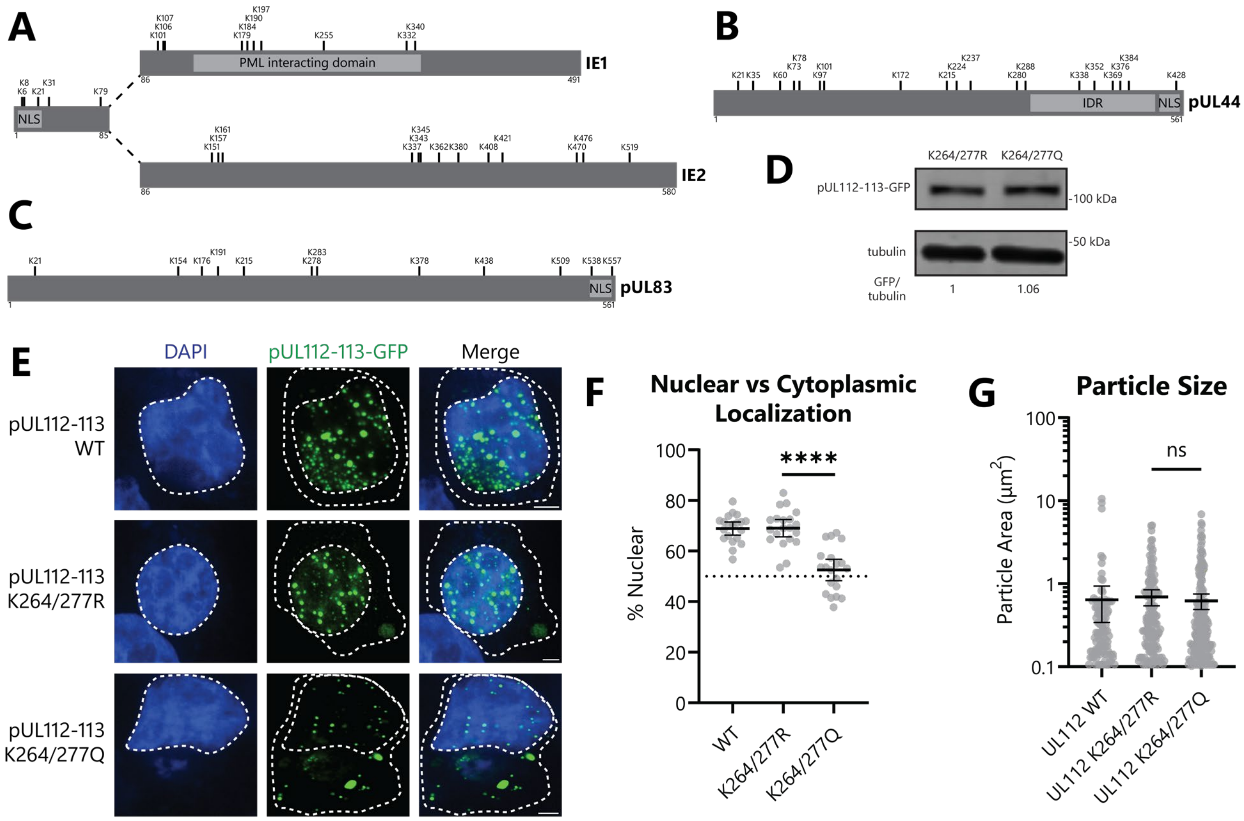

Fig. S3. Lactylation decorates many HCMV proteins. (**A** to **C**) Schematic of identified lactyllysines on the immediate early viral proteins IE1 and IE2 (splice variants with a shared first 85 amino acids), delayed early protein pUL44, and late protein pUL83. (**D**) Immunoblot stained with anti-GFP and anti-tubulin (loading control). Blot shows similar expression levels between mutagenized K264/277R and K264/277Q pUL112-113-GFP constructs. (**E** and **F**) Transfection of plasmid expressing WT, K264/277R, or K264/277Q pUL112-113-GFP in HEK293T cells. Representative images at 100× are shown (scale bar = 2.5 µm). % Nuclear calculated by percent of total cellular fluorescence intensity within the nucleus (10 nuclei per *n*; *n* = 3). (**G**) Quantification of UL112-GFP particle size in transfected cells (10 cells per condition; ~200 total particles per condition). Bar plots are mean ± 95% CI, with significance determined by two-tailed Student's *t* test (F and G). ns = not significant, ****$P < 0.0001$.

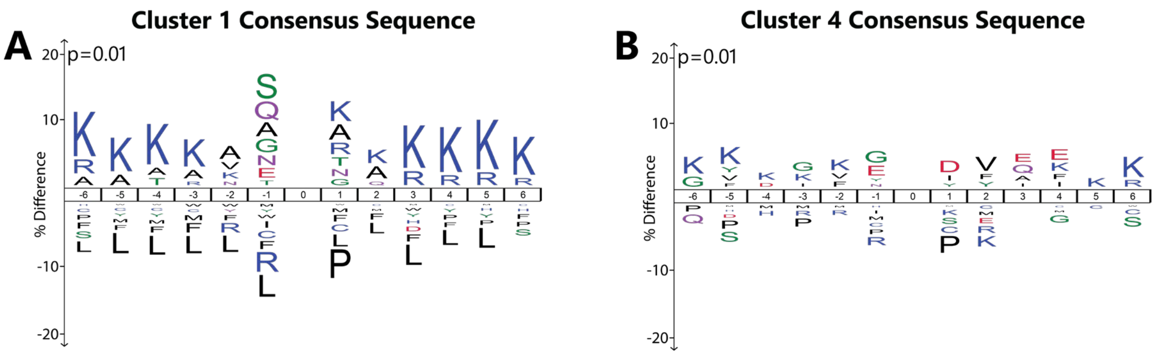

Fig. S4. Lactyllysine consensus sequences across temporal abundance clusters. (**A**) Lactyllysine consensus sequences for Cluster 1 or (**B**) Cluster 4 host lactyllysine sites determined with iceLogo. Sites in Cluster 4 were randomly re-sampled to equalize the number of sites in Cluster 1 (*n* = 3,026) prior to consensus enrichment analysis.

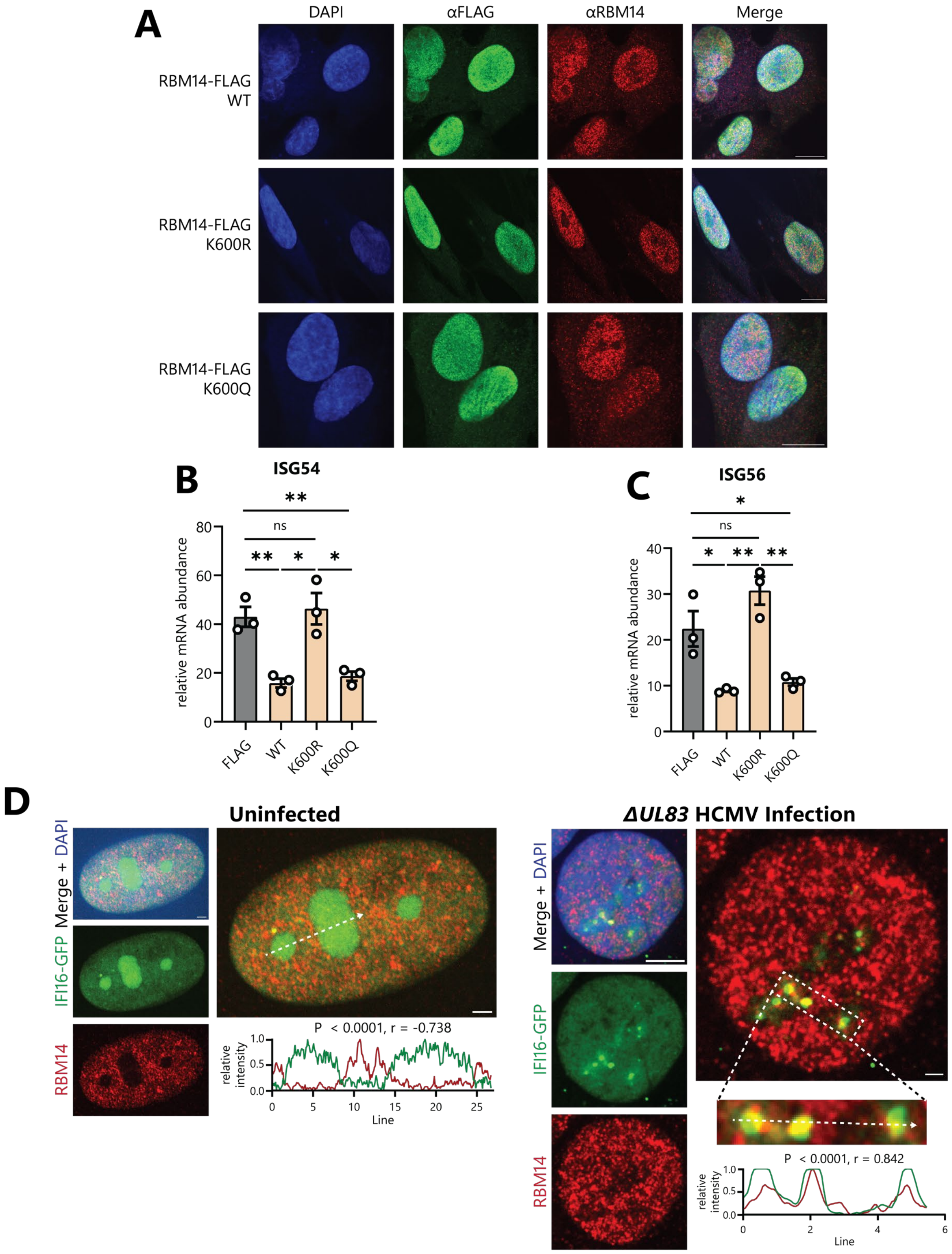

A
DAPI
αFLAG
αRBM14
Merge
RBM14-FLAG WT
RBM14-FLAG K600R
RBM14-FLAG K600Q
B
ISG54
relative mRNA abundance
FLAG
WT
K600R
K600Q
ns
C
ISG56
D
Uninfected
ΔUL83 HCMV Infection
Merge + DAPI
IFI16-GFP
RBM14
P < 0.0001, r = -0.738
P < 0.0001, r = 0.842
relative intensity
Line

Fig. S5. RBM14 regulates innate immune signaling. (**A**) MRC-5 cell lines expressing RBM14-FLAG WT, K-to-R (charge-mimic), and K-to-Q (lactyl-mimic) constructs. Cells were stained for FLAG and RBM14. Representative images at 100× are shown (scale bar = 10 µm). Cell lines show the expected nuclear localization for endogenous RBM14 and FLAG-tagged constructs. (**B** and **C**) *ISG54* and *ISG56* mRNA levels were quantified by qPCR (ΔΔCt against GAPDH) (HCMV MOI 5, 6 HPI, n = 3). Replicates were normalized by the average FLAG mRNA levels. (**D**) MRC-5 fibroblasts expressing IFI16-GFP were left uninfected or infected with *ΔUL83* HCMV (MOI 5, 6HPI), then stained for endogenous RBM14. Representative images at 100× are shown (scale bar = 2.5 µm). Colocalization (PCC) between IFI16-GFP and RBM14 was measured at the line (10 nuclei per n; n = 3). Bar plots are mean ± SEM, with significance determined by two-tailed Student's t test (B and C). ns = not significant, *$P < 0.05$, and **$P < 0.01$.

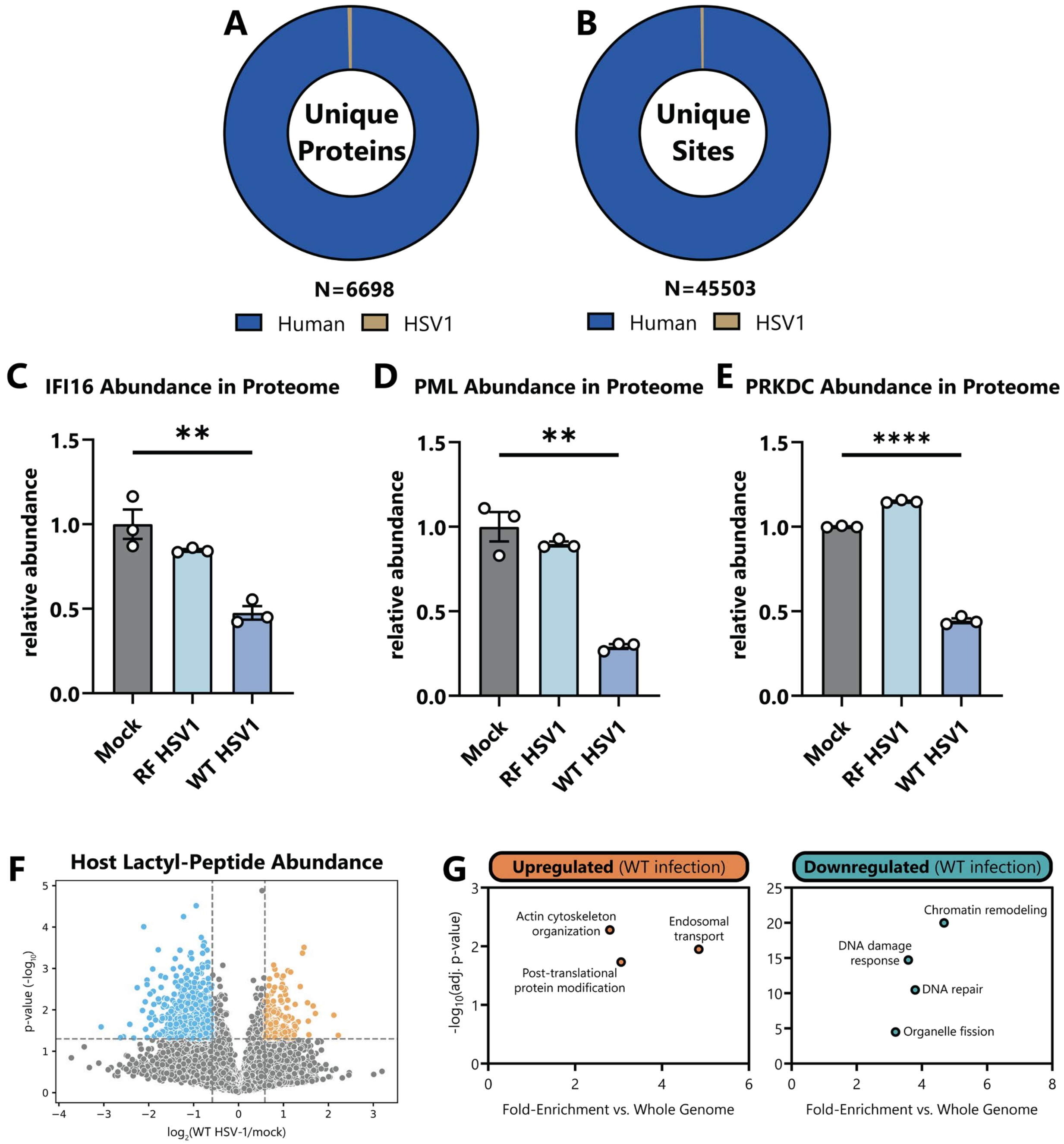

Fig. S6. Overview of global proteome lactylation dataset during HSV-1 infection. (**A**) Number of unique lactyllysine sites identified across (**B**) unique host and viral proteins in the HSV-1 global lactylome dataset. (**C** to **E**) Abundance of IFI16, PML, and PRKDC in the whole cell proteome during mock (uninfected), RF (*ICP0-RF*), or WT HSV-1 infection at 6 hours post-infection. (**F**) Volcano plot showing host protein lactyl-peptide abundances (normalized to protein abundance) as $\log_2$ fold-change over mock abundance during WT HSV-1 infection. Dotted lines show threshold for differentially regulated sites: fold change = 1.5 and $P < 0.05$. (**G**) Scatter plots showing select enriched gene ontology (GO) biological processes among upregulated or downregulated lactyl-peptides using g:Profiler. Bar plots are mean ± SEM, with significance

determined by two-tailed Student's *t* test (A and B). *$P < 0.05$, **$P < 0.01$, ***$P < 0.001$, and ****$P < 0.0001$.

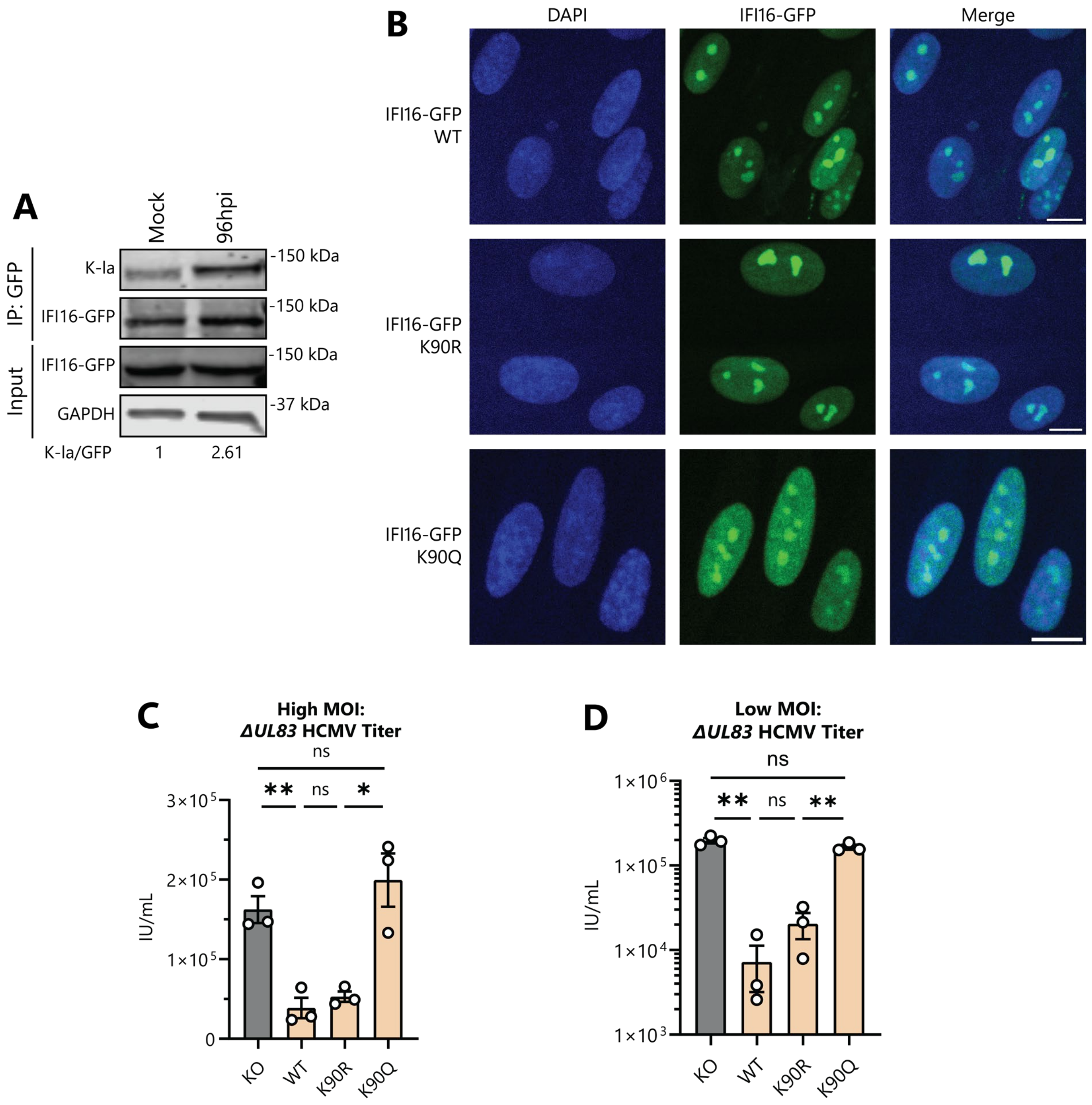

Fig. S7. IFI16 lactylation upon HCMV infection supports viral replication and spread. (**A**) IFI16-GFP cells were either mock infected or infected with HCMV (MOI 3) and collected at 96 hours post-infection (hpi), then immunoprecipitated (IP) with anti-GFP antibody beads before using anti-lactyllysine antibody to determine changes in IFI16 lactylation during infection. Anti-GFP and anti-GAPDH staining shows equal loading in the input and after the IP. This blot is representative of three biological replicates. (**B**) MRC-5 cell lines expressing IFI16-GFP WT, K-to-R (charge-mimic), and K-to-Q (lactyl-mimic) constructs. IFI16-GFP constructs show apparent nuclear and nucleolar localization, agreeing with expected IFI16 localization in WT fibroblasts. Representative images at 100× are shown (scale bar = 10 μm). (**C**) Virus titer after *ΔUL83* HCMV infection of IFI16 cell lines or KO cell line (*ΔIFI16*; control) at MOI 1 with collection at 5 DPI (n = 3) or (**D**) MOI 0.01 with collection at 12 DPI (n = 3); IU/mL = infectious units per mL. ns = not significant, *$P < 0.05$, and **$P < 0.01$.

Table S1. (separate file)

Spreadsheet containing temporal whole cell proteome data for host and viral proteins during HCMV infection of WT MRC-5 fibroblasts.

Table S2. (separate file)

Spreadsheet containing temporal lactyl-peptide data for host and viral proteins during HCMV infection of WT MRC-5 fibroblasts. Also contains enriched GO biological processes and CORUM complexes across clustered lactyl-peptides (related to Fig. 4) and comparison of the number of lactylation sites identified to PaxDB absolute protein abundance (related to Fig. 1).

Table S3. (separate file)

Spreadsheet containing whole cell proteome data for host and viral proteins during WT or *ICP0-RF* HSV-1 infection of WT MRC-5 fibroblasts at 6 hours post-infection.

Table S4. (separate file)

Spreadsheet containing lactyl-peptide data for host and viral proteins during WT or *ICP0-RF* HSV-1 infection of WT MRC-5 fibroblasts at 6 hours post-infection. Also contains enriched GO biological processes among differentially regulated lactyl-peptides (related to Figs. 6 and S6).

Table S5. (separate file)

Spreadsheet containing whole cell proteome data for host proteins during HCMV infection of MRC-5 fibroblasts at 96 hours post-infection. Cells were treated for the duration of the infection with either 100mM alanine or a shRNA KD targeting the AARS1 lactyltransferase.

Table S6. (separate file)

Spreadsheet containing lactyl-peptide data for host proteins during HCMV infection of MRC-5 fibroblasts at 96 hours post-infection. Cells were treated for the duration of the infection with either 100mM alanine or a shRNA KD targeting the AARS1 lactyltransferase.

Table S7. (separate file)

Spreadsheet containing all data values for figures with a small sample size ($N < 20$).
